# Supplementary material for: Ligand-induced conformational changes in the β1-adrenergic receptor revealed by hydrogen-deuterium exchange mass spectrometry
Source: Nat Commun. 2024 Oct 18;15:8993. doi: 10.1038/s41467-024-53161-0 (PMC11489754; doi:10.1038/s41467-024-53161-0)
Supplement: Supplementary file 1 — Supplementary Information [file 41467_2024_53161_MOESM1_ESM.pdf]

Supplementary Information for

Ligand-induced conformational changes in β1-Adrenergic Receptor Revealed by Hydrogen-Deuterium Exchange Mass Spectrometry

Joanna Toporowska[1]; Parth Kapoor[2]; Maria Musgaard[2], Karolina Gherbi[2], Kathy Sengmany[2], Feng Qu[2], Mark Soave[2], Hsin-Yung Yen[2], Kjetil Hansen[1]; Ali Jazayeri[2]; Jonathan T.S. Hopper[2, *]; Argyris Politis[3,4, *]

[1]King's College London, London, United Kingdom
[2]OMass Therapeutics, Oxford, United Kingdom
[3] Faculty of Biology, Medicine and Health, School of Biological Sciences, The University of Manchester, Manchester, UK
[4]Manchester Institute of Biotechnology, The University of Manchester, Princess Street, Manchester M1 7DN, UK

Corresponding authors: argyris.politis@manchester.ac.uk and Jonathan.hopper@omass.com

Table of content

1. **Supplementary Methods ……………………………………………………………….…. 3**
   1.1. β1AR quench buffer optimisation ……………………………………………………... 3
2. **Supplementary Figures ……………..……………….…………………………………. 4**
   2.1. Schematic representation of the sequence coverage optimisation steps ………………… 4
   2.2. Peptide maps obtained for β1AR and miniGs ………………………………………..… 5
   2.3. Chemical structures of ligands used in HDX experiments ………………………………….. 6
   2.4. Isoprenaline and noradrenaline concentration response curves for cAMP accumulation 6
   2.5. Western blot analysis of membrane preparations of CHO cells …………………………… 7
   2.6. Heat map of tβ1AR apo state ………………………………………………………………….. 7
   2.7. Woods plots for all HDX experiments ………………………………………………….... 8
   2.8. Graphical visualisation of the results obtained from the HDX experiments ……………... 18
   2.9. Uptake plots for all observed HDX effects in every experiment ………………………..… 25
3. **Supplementary Tables ………………………………...…………………………………… 38**
   3.1. Results for β1AR quench buffer optimisation……………………………………………….. 38
   3.2. Ligand binding occupancies ……………….…………………………..…………….….. 39
   3.3. Summary of all observed effects for β1AR in complex with tested ligands …………….….. 39
   3.4. HDX-MS data tables ……………………………………………………………………..… 40
4. **Supplementary References ….....………………………………………………………..... 46**

1. Supplementary Methods.

1.1. Quench buffer optimisation.

The optimization process began by screening various compositions of quench buffers, starting with a composition of 50 mM KH_2PO_4, 50 mM K_2HPO_4, and 0.02% DDM. The aim was to determine the most efficient conditions for the digestion process; therefore, two types of pepsin columns were employed: the Waters EnzymateTH BEH pepsin column and a self-packed pepsin column with Immobilized Pepsin Agarose. Initial trials with the Waters column yielded a sequence coverage of less than 50%. Subsequent replacement with the self-packed pepsin column led to a notable increase in coverage to 75%. Additionally, increasing the DDM concentration in the quench buffer from 0.01% to 0.1%, exceeding the documented CMC, resulted in a significant 20% improvement in coverage. To address presence of disulfide bonds in β1AR, the reducing agent TCEP (tris(2-carboxyethyl)phosphine hydrochloride) was incorporated into the quench buffer. Utilizing both normal collision energy (CE) and look-up table (LUT) collision energy, along with 100 mM TCEP, raised the coverage to nearly 90%. Further optimization steps included modifying the LC-MS method to enhance peak resolution by adjusting elution times and gradients (8-70% B, 8-55% B). Prolonging the sample's retention on the pepsin column for slow digestion and introducing the enzyme *Rhizopus.sp* to the quench buffer were also implemented. Both C18 and C8 analytical columns were tested. The final optimization step involved using a dual protease type XIII/pepsin column. This change significantly boosted sequence coverage to approximately 94%, with a redundancy of 8.27.

2. Supplementary Figures.

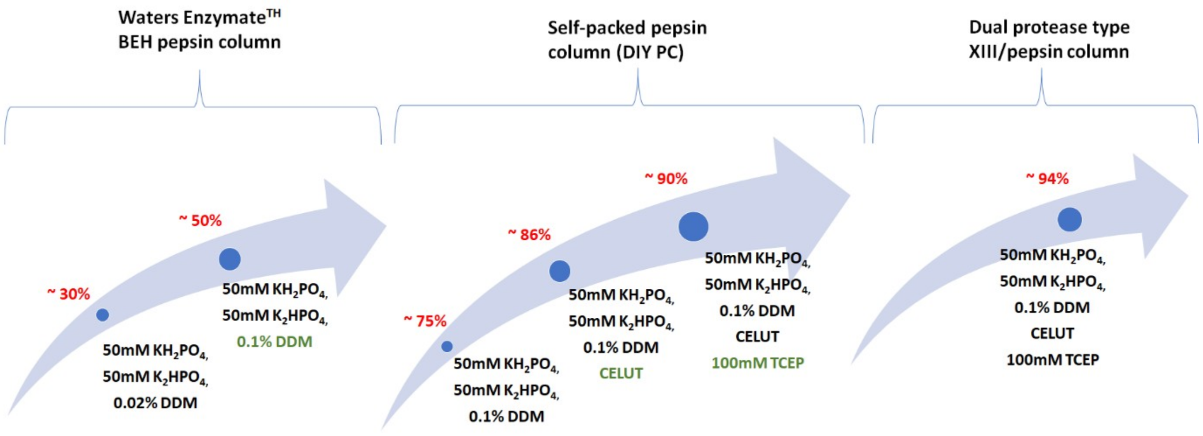

Supplementary Figure 1. Schematic representation of the sequence coverage optimisation steps. The diagram depicts the optimization process for the tβ1-adrenergic receptor (tβ1AR). It outlines the steps undertaken to enhance sequence coverage using three distinct columns: the Waters EnzymateTH BEH pepsin column, a self-packed pepsin column (referred to as DIY PC), and the dual protease type XIII/pepsin column.

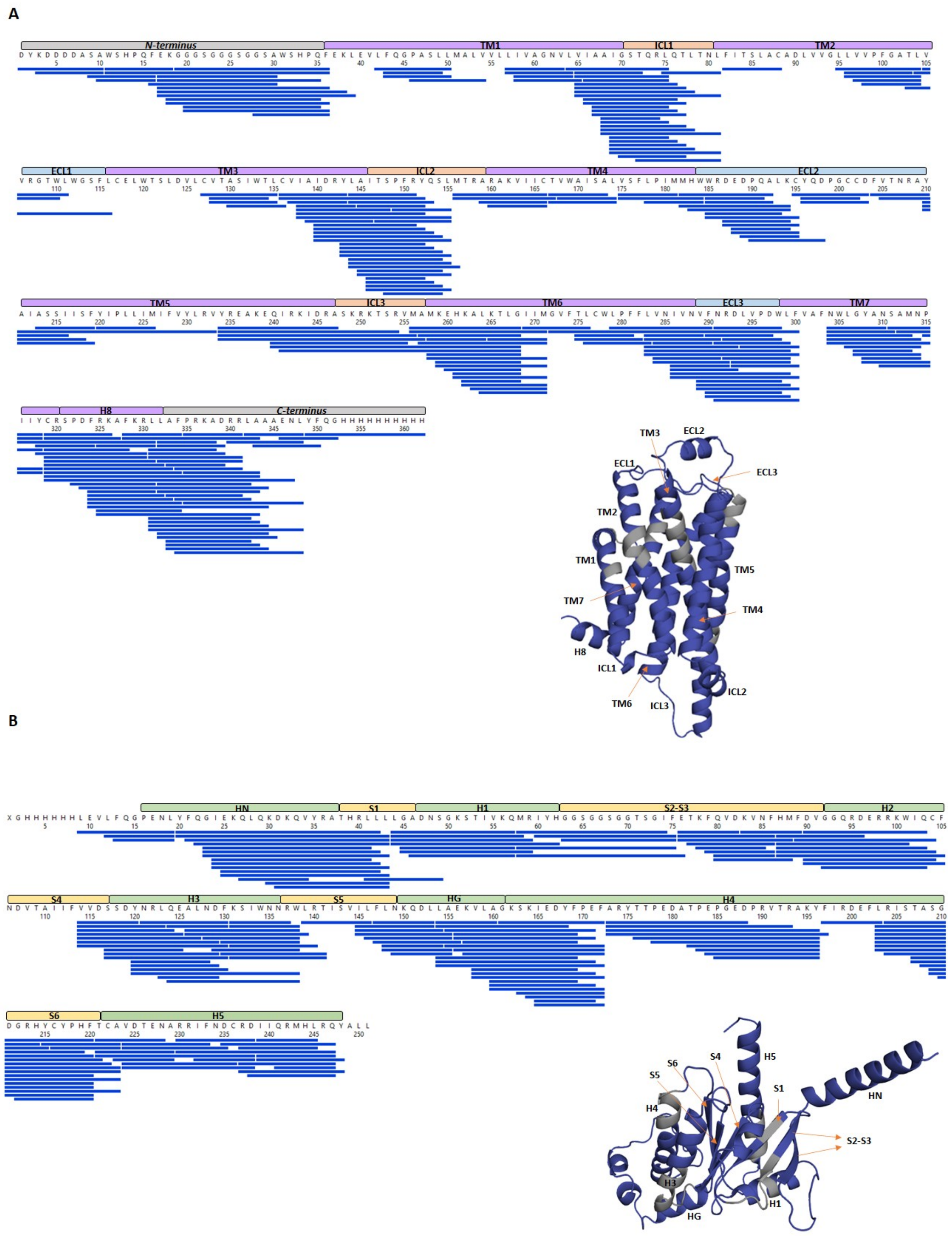

Supplementary Figure 2. Peptide maps obtained for tβ1AR and miniGs. **A** tβ1AR sequence coverage with displayed number of peptides covering parts of the protein. Achieved coverage: 93.6%, redundancy: 8.27. **B** miniGs sequence coverage 92.4% and redundancy: 7.26. Results are visualised in PyMOL. Blue colour indicates covered areas of the proteins.

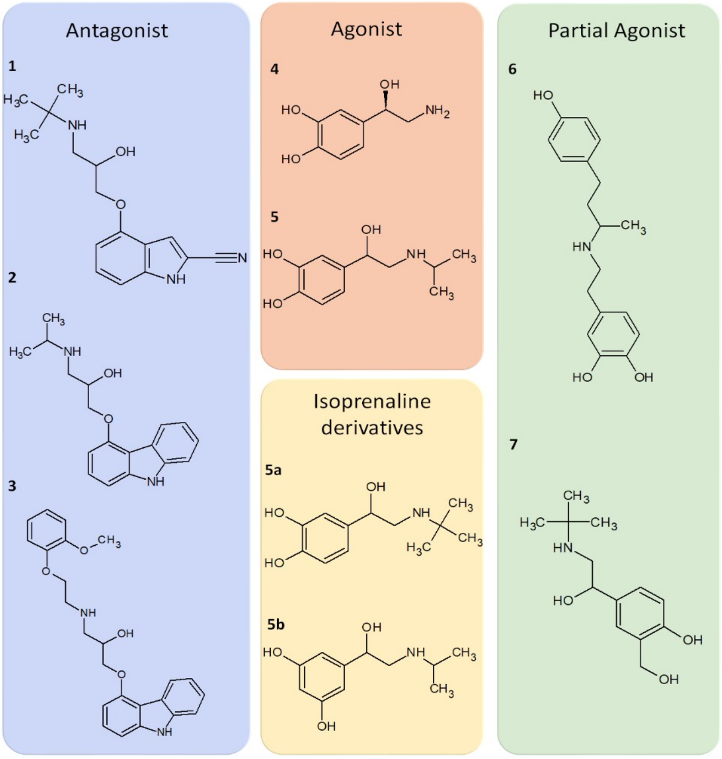

Supplementary Figure 3. Chemical structures of ligands used for analysis. Antagonists: **1** Cyanopindolol, **2** Carazolol, **3** Carvedilol. Agonists: **4** Norepinephrine, **5** Isoprenaline. Partial Agonists: **6** Dobutamine, **7** Salbutamol. Isoprenaline derivatives: **5a** Colterol Hydrochloride, **5b** Orciprenaline.

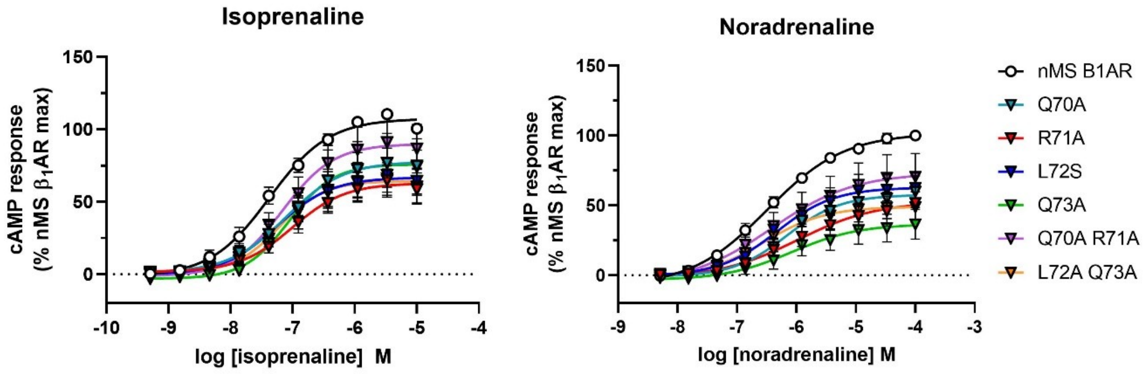

Supplementary Figure 4. Isoprenaline and noradrenaline concentration response curves for cAMP accumulation determined in CHO cells transiently transfected with either wild type (nMS) and other tβ1AR constructs. Data are expressed as mean ± SEM of 3-11 independent experiments performed in duplicate. Error bars not shown lie within the dimensions of the symbol.

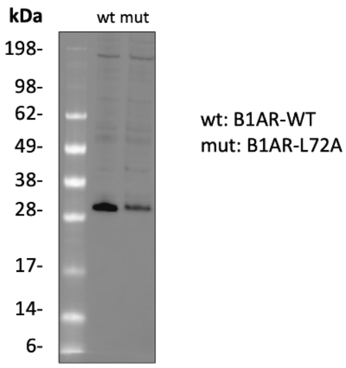

Supplementary Figure 5. Western blot analysis of membrane preparations of CHO cells transiently transfected with either wild type or L72 β1AR confirmed expression of both receptor variants. Data shown are a representative of 2 independent experiments.

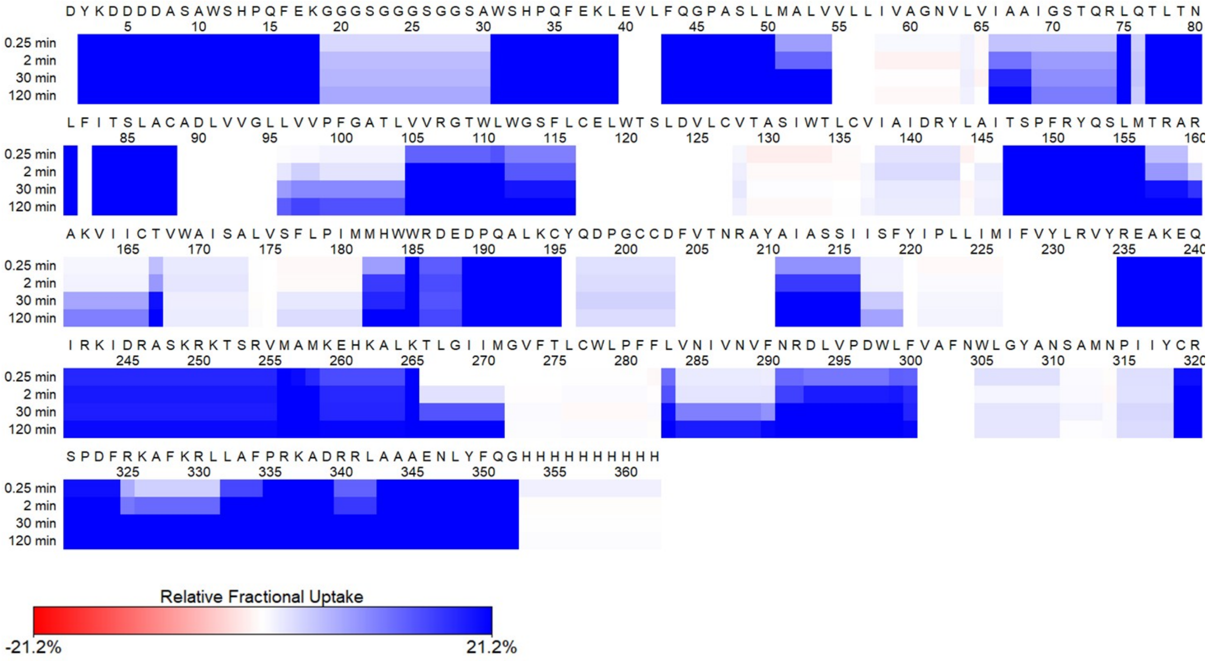

Supplementary Figure 6. Heat map of tβ1AR apo state. The snapshot of the deuterium uptake profile of the tβ1AR in its unbound form, reflecting on the protein's folding landscape and dynamics.

Supplementary Figures (7-11)

Woods plots for all HDX experiments.

tβ1AR apo vs tβ1AR + Carazolol

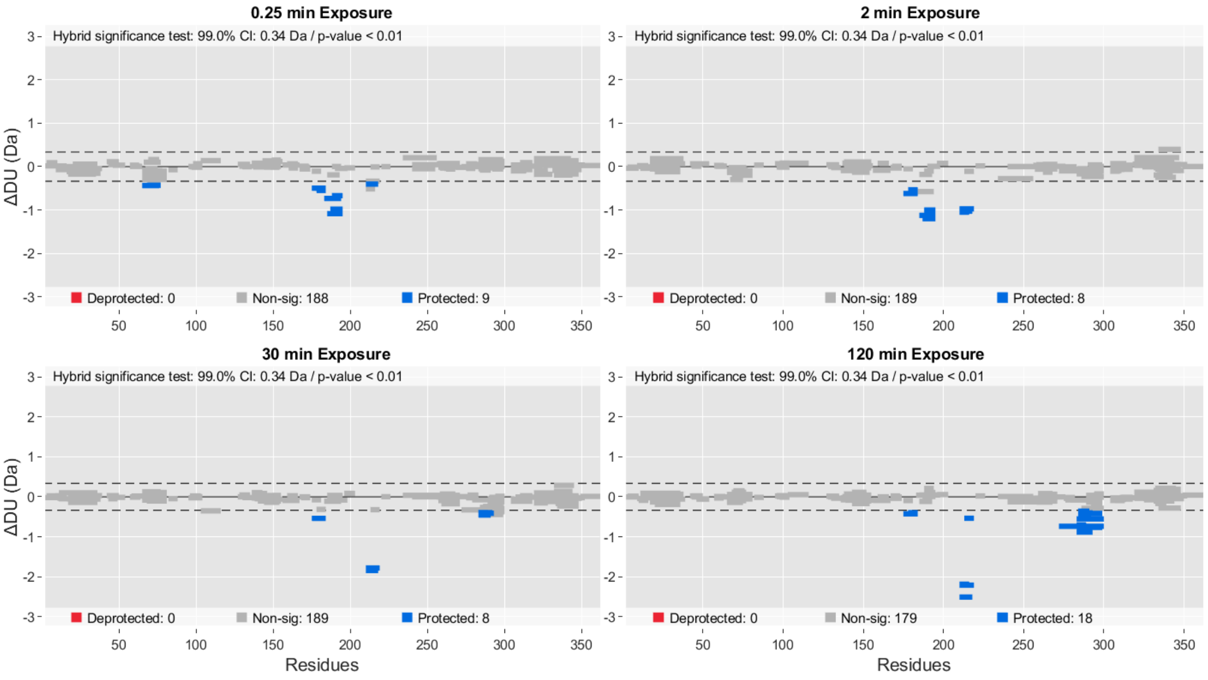

tβ1AR apo vs tβ1AR + Carvedilol

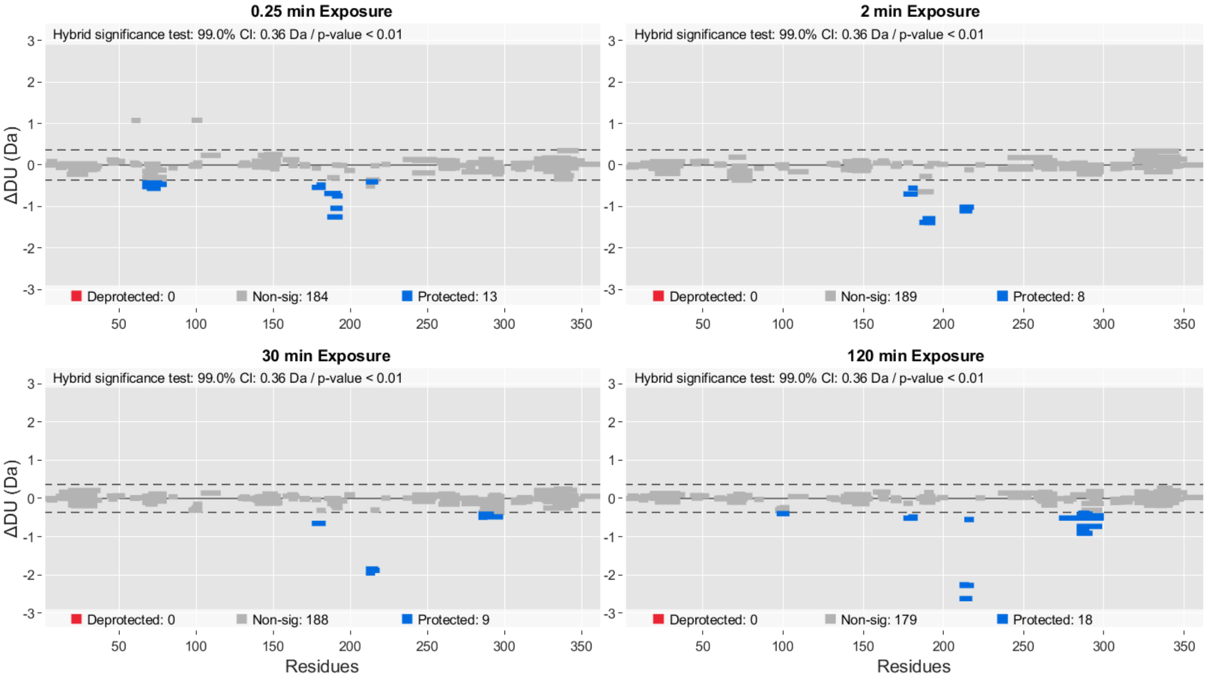

tβ1AR apo vs tβ1AR + Cyanopindolol

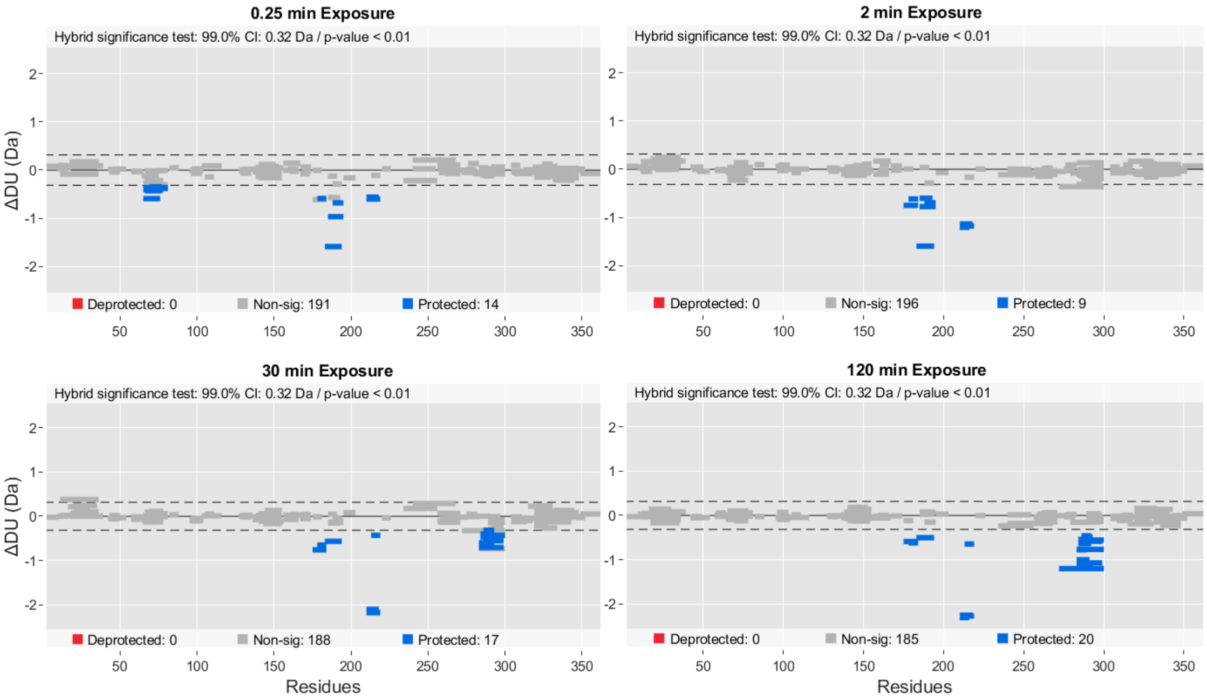

tβ1AR apo vs tβ1AR + Isoprenaline

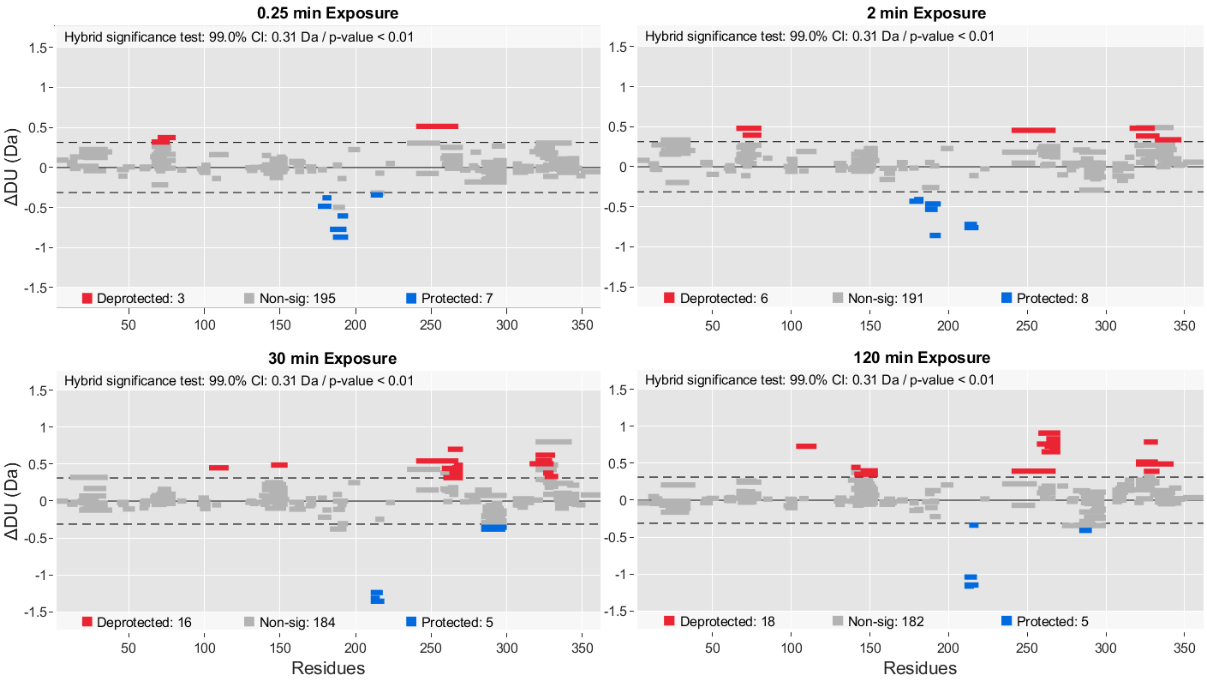

tβ1AR apo vs tβ1AR + Norepinephrine

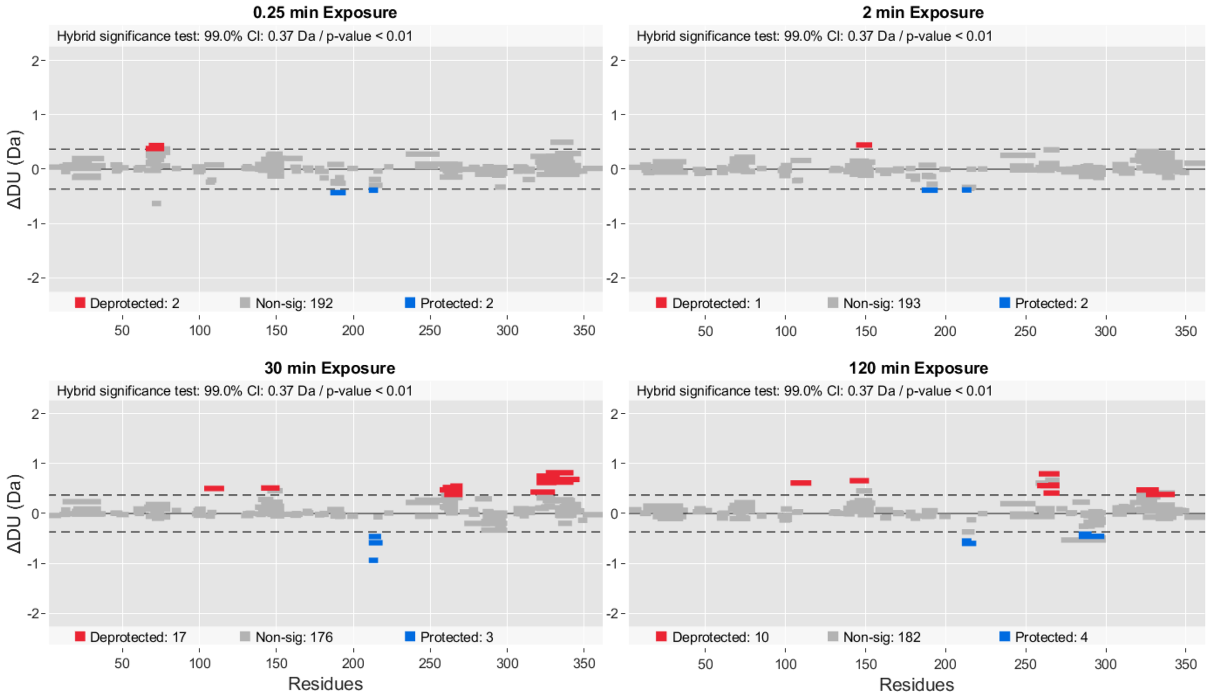

tβ1AR apo vs tβ1AR + Dobutamine

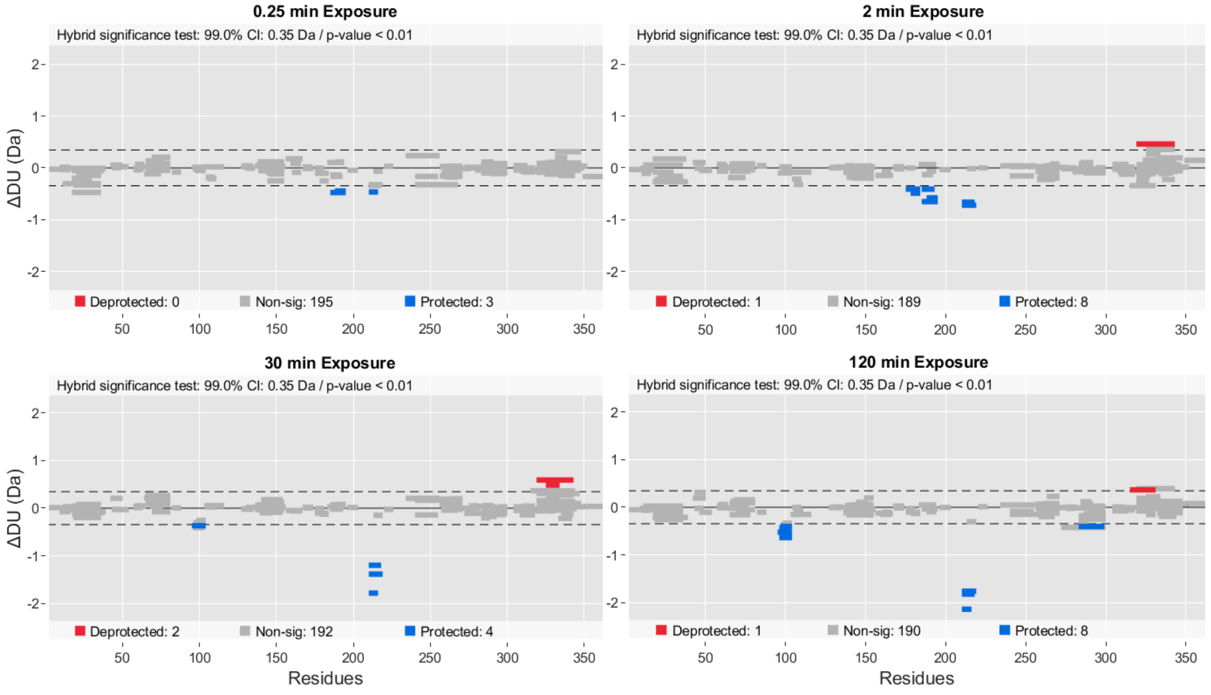

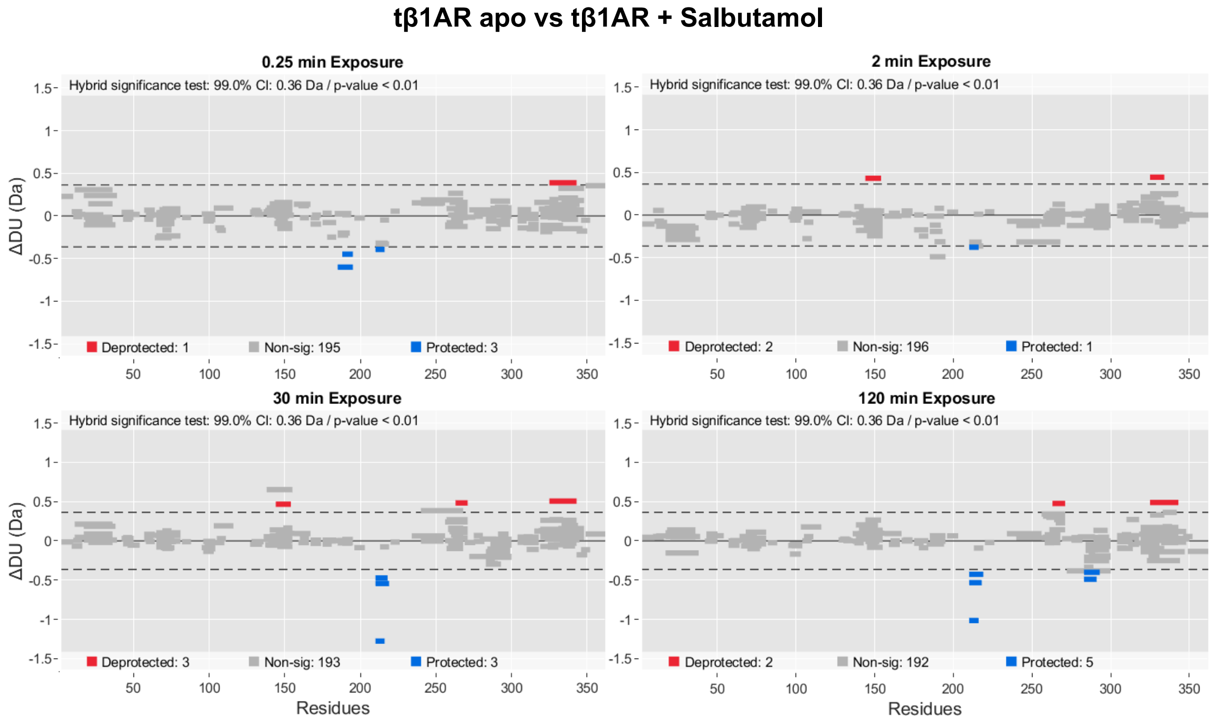

Supplementary Figure 7. HDX Experiment 1,2 and 3: tβ1AR with ligands (agonist, partial agonist, antagonist). Deuterium uptake differences presented in Woods plot, which provides breakdown of peptides ensemble for 0.25-, 2-, 30- and 120-min time point. It displays peptide length, global coverage, and deuterium uptake. 99% confidence limit was applied to the data set to identify peptides with significant deuterium uptake. Deprotected, protected and non-significantly different peptides are in red, blue, and grey respectively. Presented plots have been created in Deuteros 2.0[33].

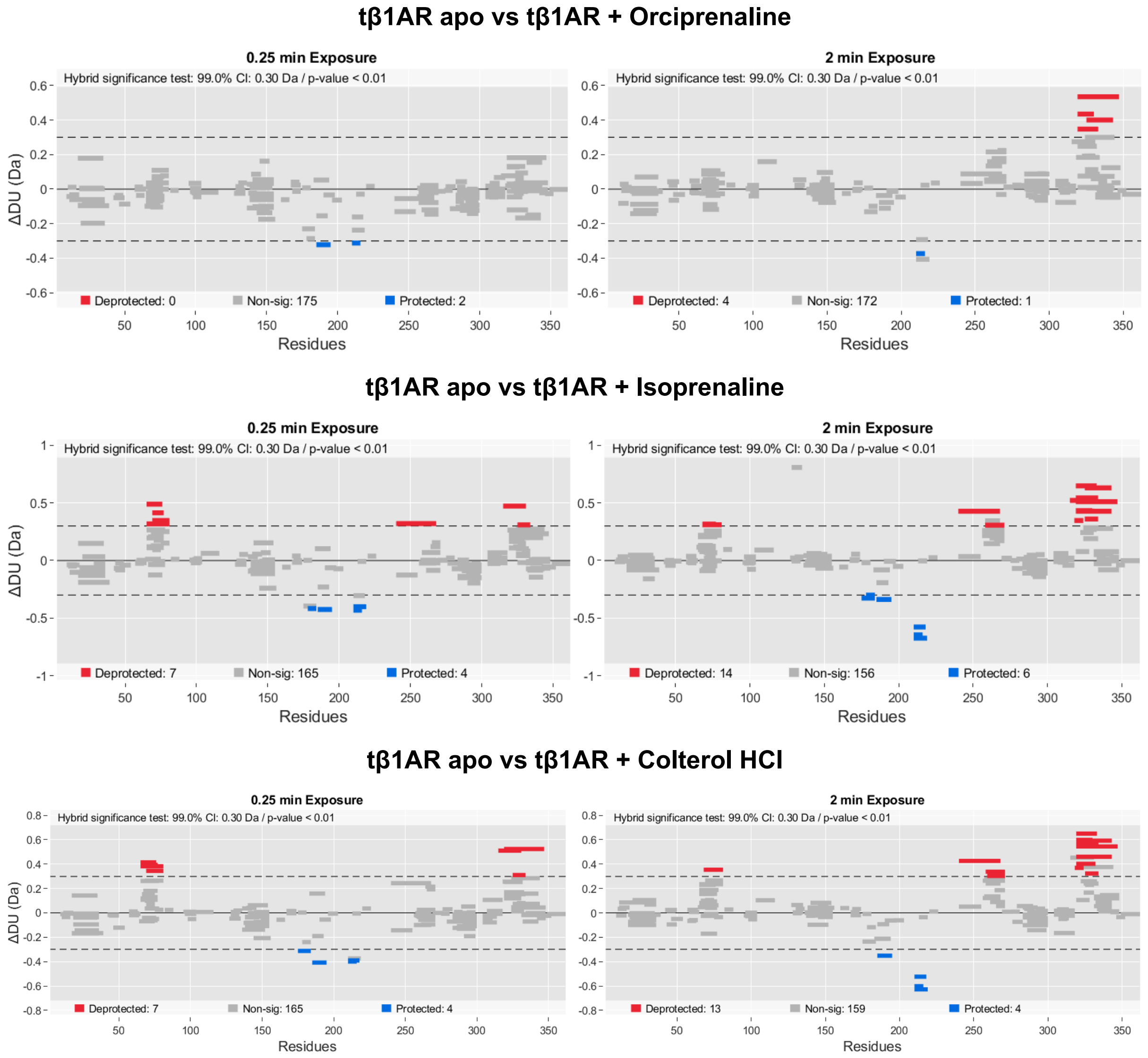

Supplementary Figure 8. HDX Experiment 4: tβ1AR with Isoprenaline derivatives. Deuterium uptake differences presented in Woods plot, which provides breakdown of peptides ensemble for 0.25- and 2-min time point. It displays peptide length, global coverage, and deuterium uptake. 99% confidence limit was applied to the data set to identify peptides with significant deuterium uptake. Deprotected, protected and non-significantly different peptides are in red, blue, and grey respectively. Presented plots have been created in Deuteros 2.0[33].

tβ1AR_L72A vs tβ1AR_L72A + Cyanopindolol

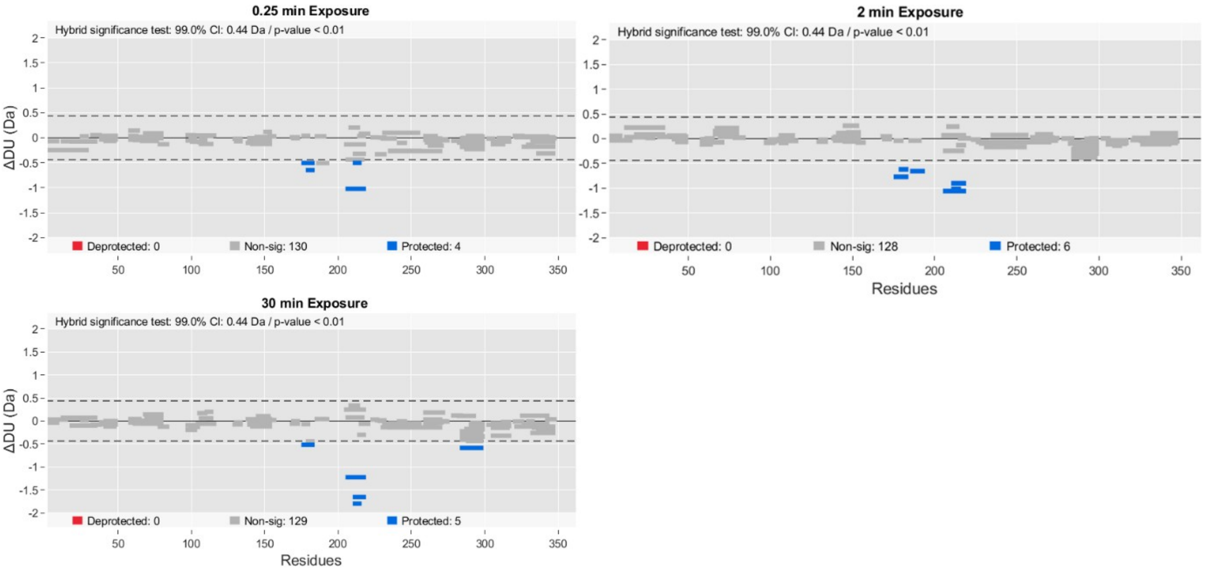

tβ1AR_L72A vs tβ1AR_L72A + Isoprenaline

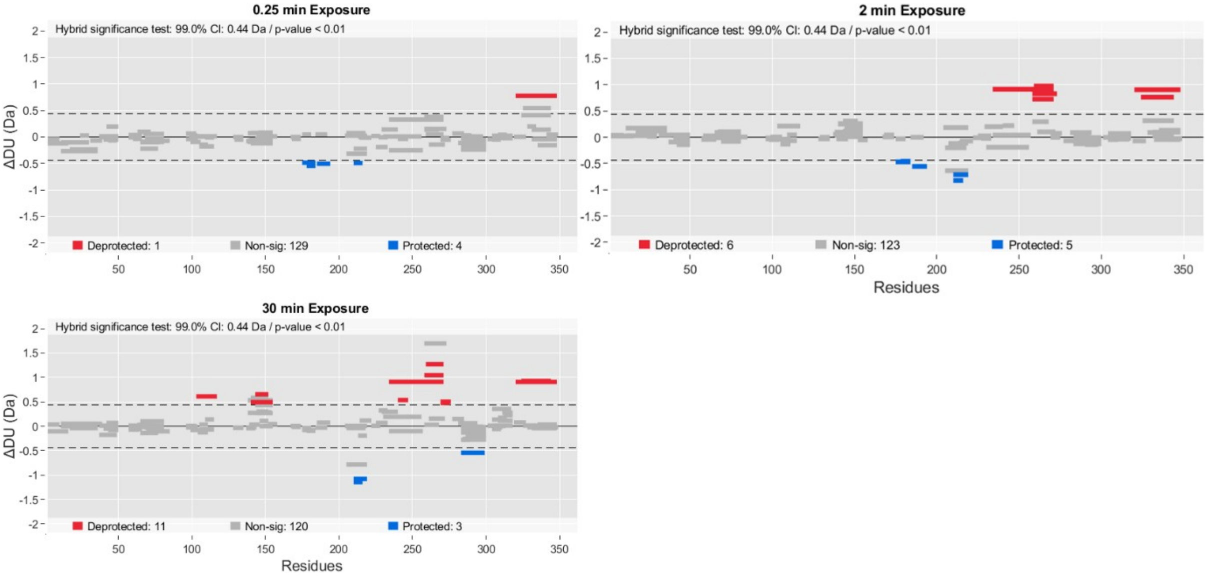

Supplementary Figure 9. HDX Experiment 5: tβ1AR_L72A mutant with Isoprenaline and Cyanopindolol. Deuterium uptake differences presented in Woods plot, which provides breakdown of peptides ensemble for 0.25-, 2- and 30- min time point. It displays peptide length, global coverage, and deuterium uptake. 99% confidence limit was applied to the data set to identify peptides with significant deuterium uptake. Deprotected, protected and non-significantly different peptides are in red, blue, and grey respectively. Presented plots have been created in Deuteros 2.0[33].

tβ1AR apo vs tβ1AR + miniGs + Isoprenaline

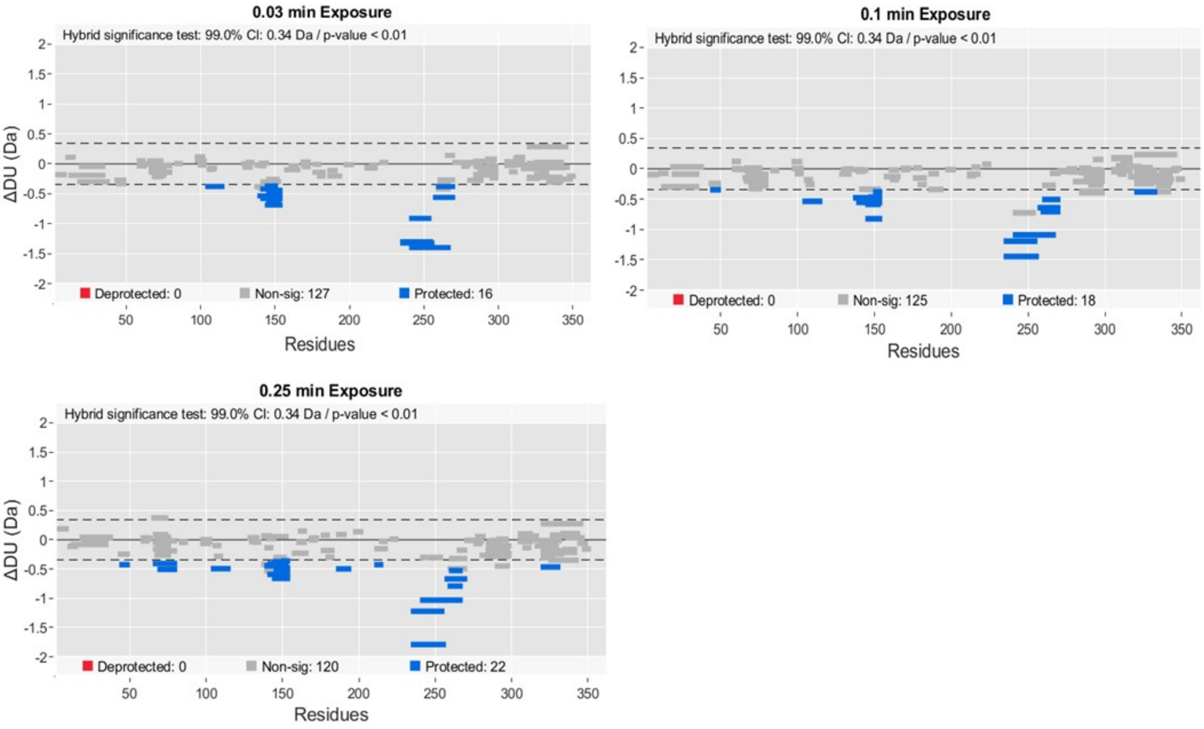

tβ1AR apo vs tβ1AR + miniGs + Dobutamine

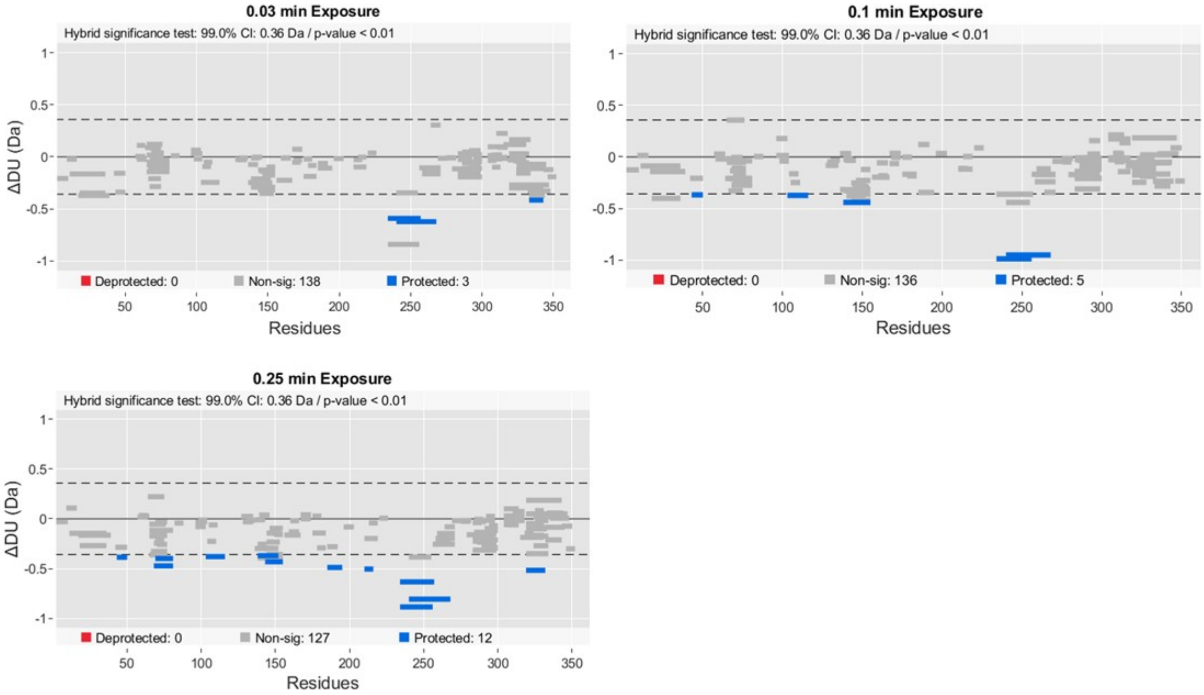

tβ1AR apo vs tβ1AR + miniGs + Cyanopindolol

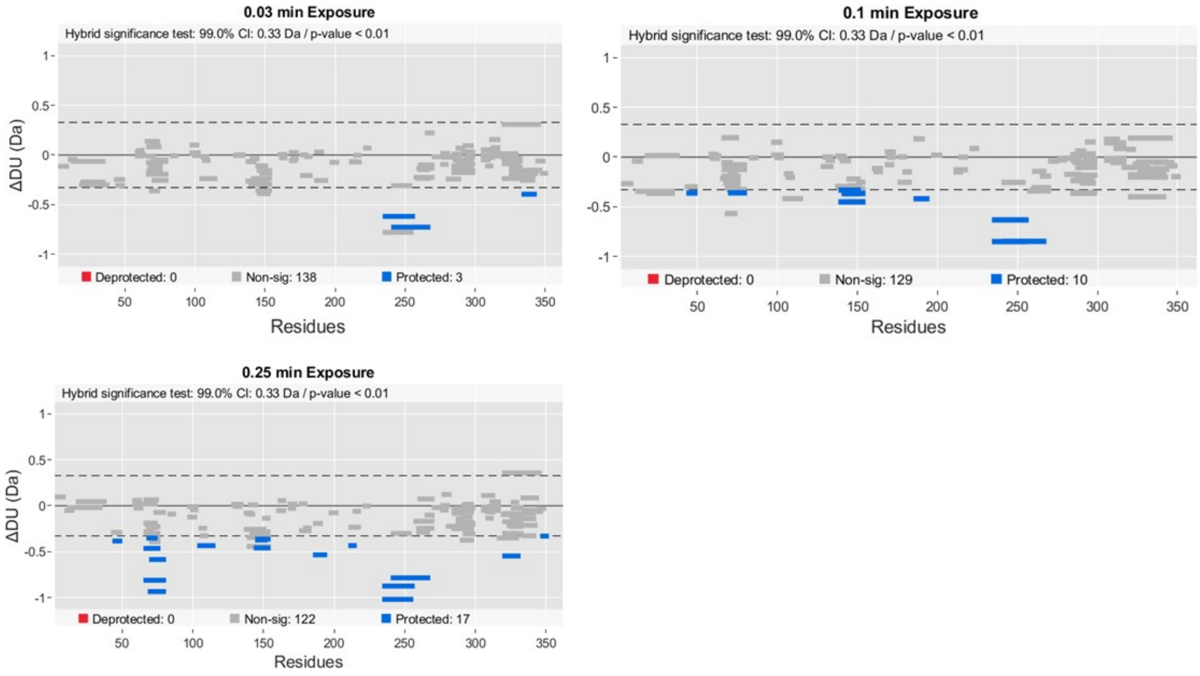

miniGs alone vs tβ1AR + miniGs + Isoprenaline

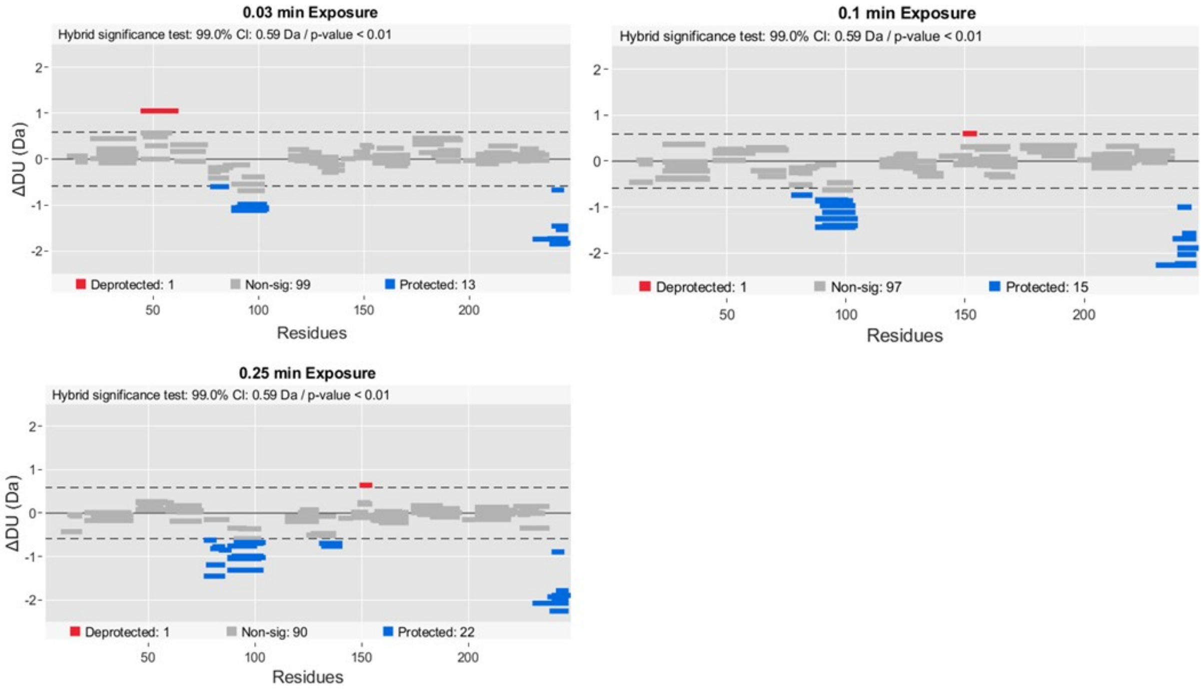

miniGs alone vs tβ1AR + miniGs + Dobutamine

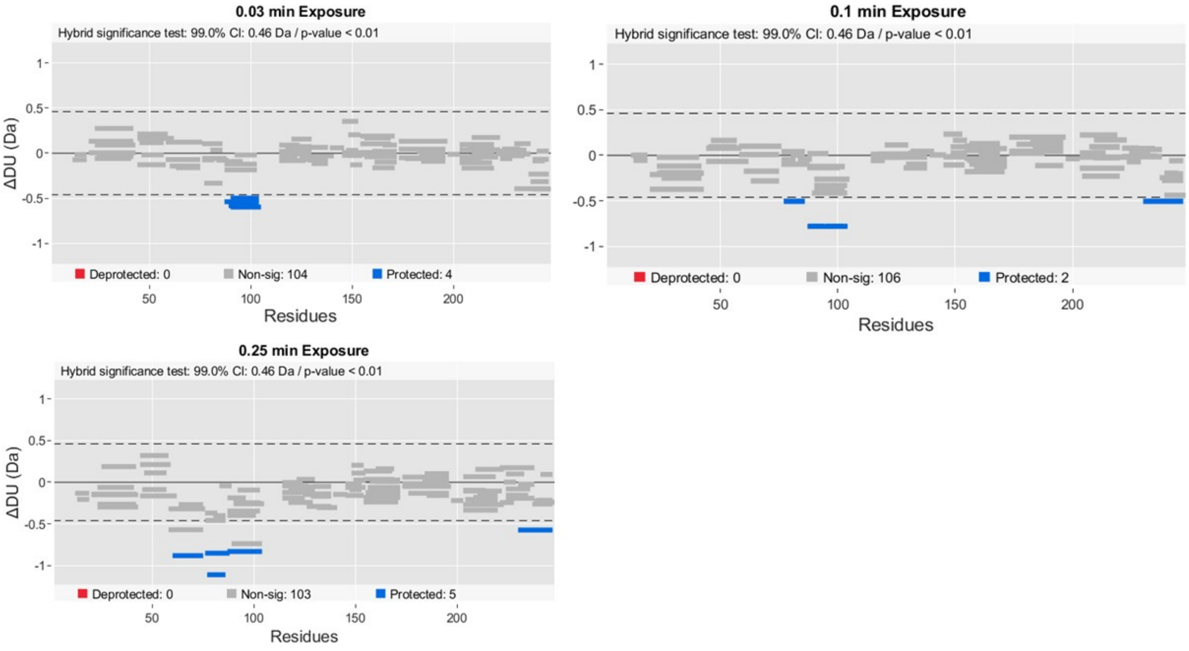

miniGs alone vs tβ1AR + miniGs + Cyanopindolol

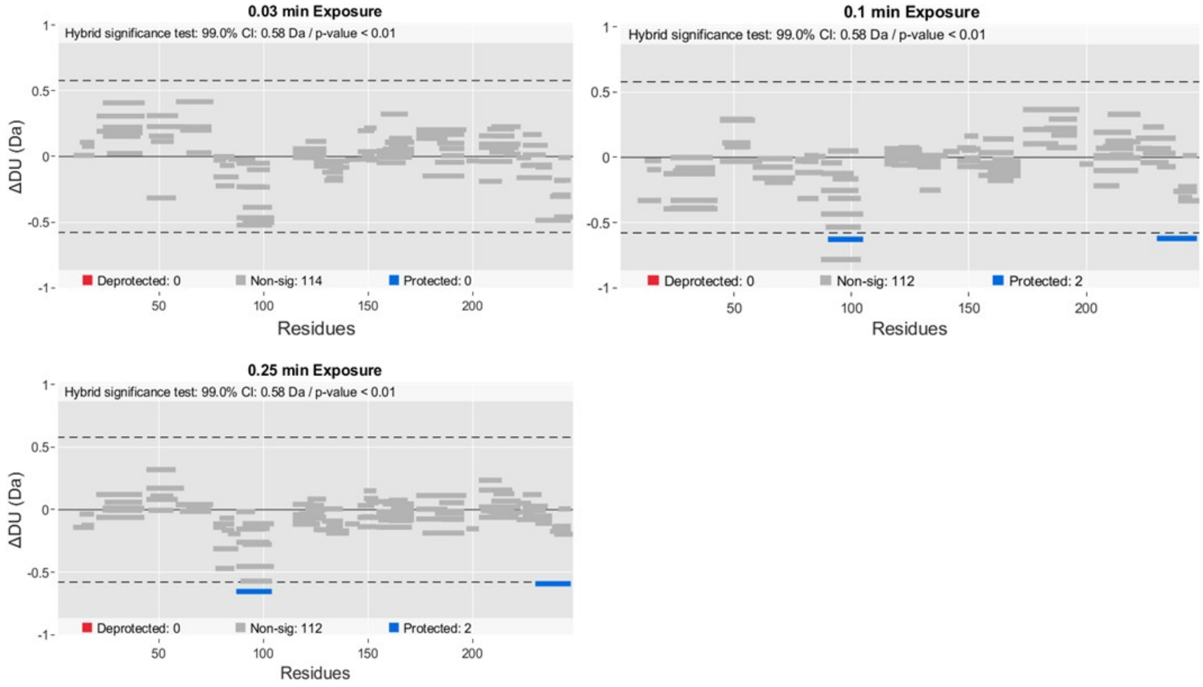

Supplementary Figure 10. HDX Experiment 6: tβ1AR in complex with miniGs and Isoprenaline, Dobutamine and Cyanopindolol. Deuterium uptake differences presented in Woods plot, which provides breakdown of peptides ensemble for 0.025-, 0.1-, 0.25 min time point. It displays peptide length, global coverage, and deuterium uptake. 99% confidence limit was applied to the data set to identify peptides with significant deuterium uptake. Deprotected, protected and non-significantly different peptides are in red, blue, and grey respectively. Presented plots have been created in Deuteros 2.0[33].

tβ1AR L72A alone vs tβ1AR L72A + miniGs + Isoprenaline

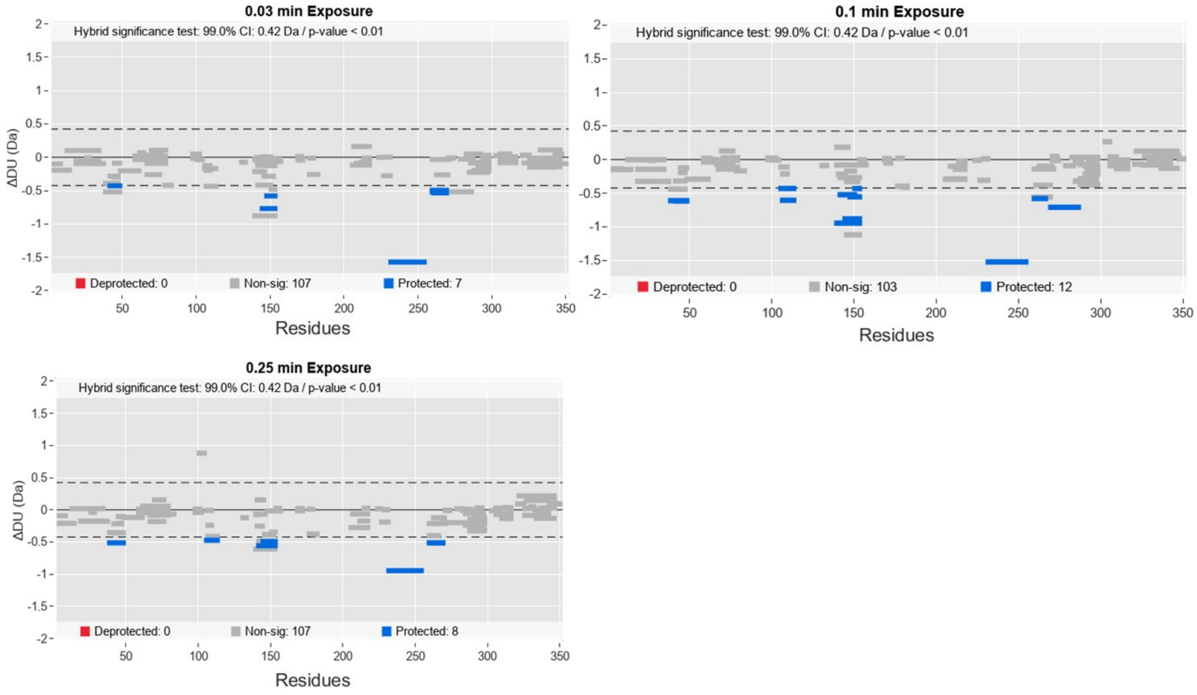

miniGs alone vs tβ1AR L72A + miniGs + Isoprenaline

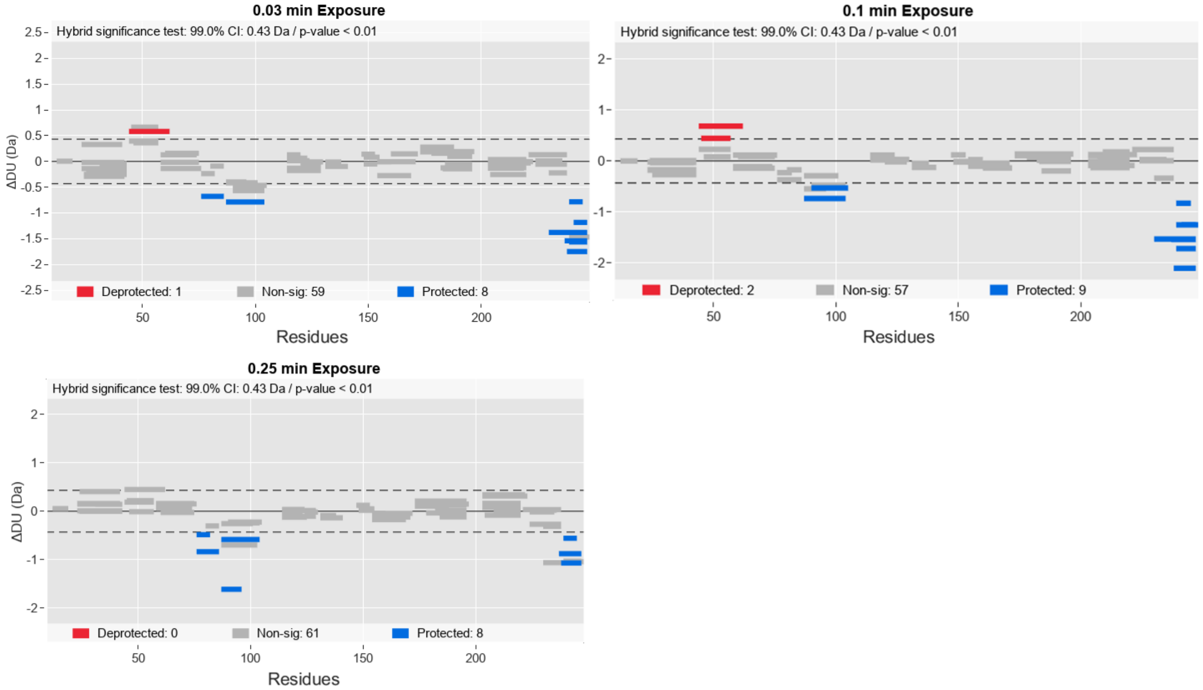

Supplementary Figure 11. HDX Experiment 7: tβ1AR L72A mutant in complex with miniGs and Isoprenaline. Deuterium uptake differences presented in Woods plot, which provides breakdown of peptides ensemble for 0.025-, 0.1-, 0.25 min time point. It displays peptide length, global coverage, and deuterium uptake. 99% confidence limit was applied to the data set to identify peptides with significant deuterium uptake. Deprotected, protected and non-significantly different peptides are in red, blue, and grey respectively. Presented plots have been created in Deuteros 2.0[33].

Supplementary Figures (12-16)

Graphical visualisation of the results obtained from the HDX experiments.

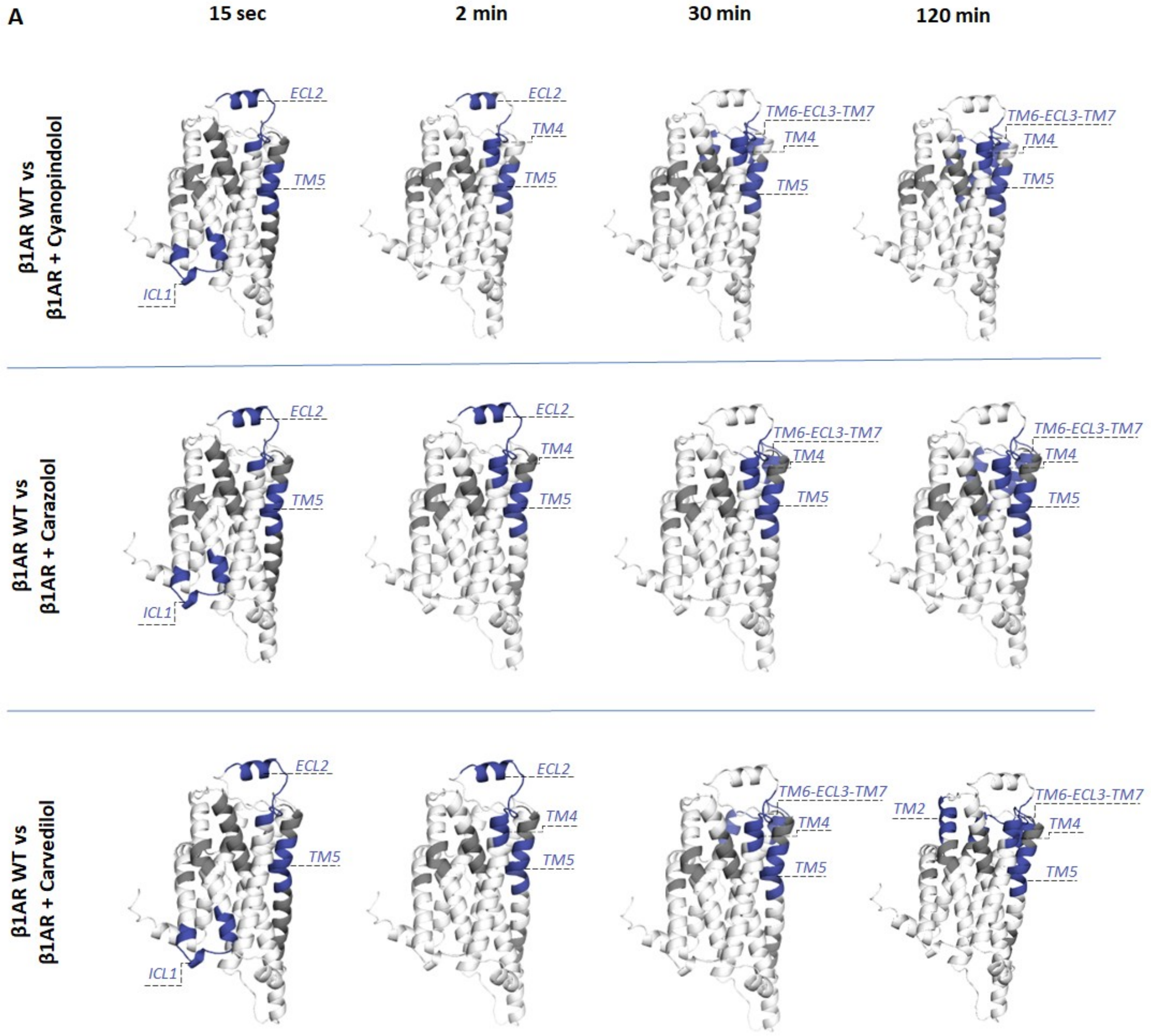

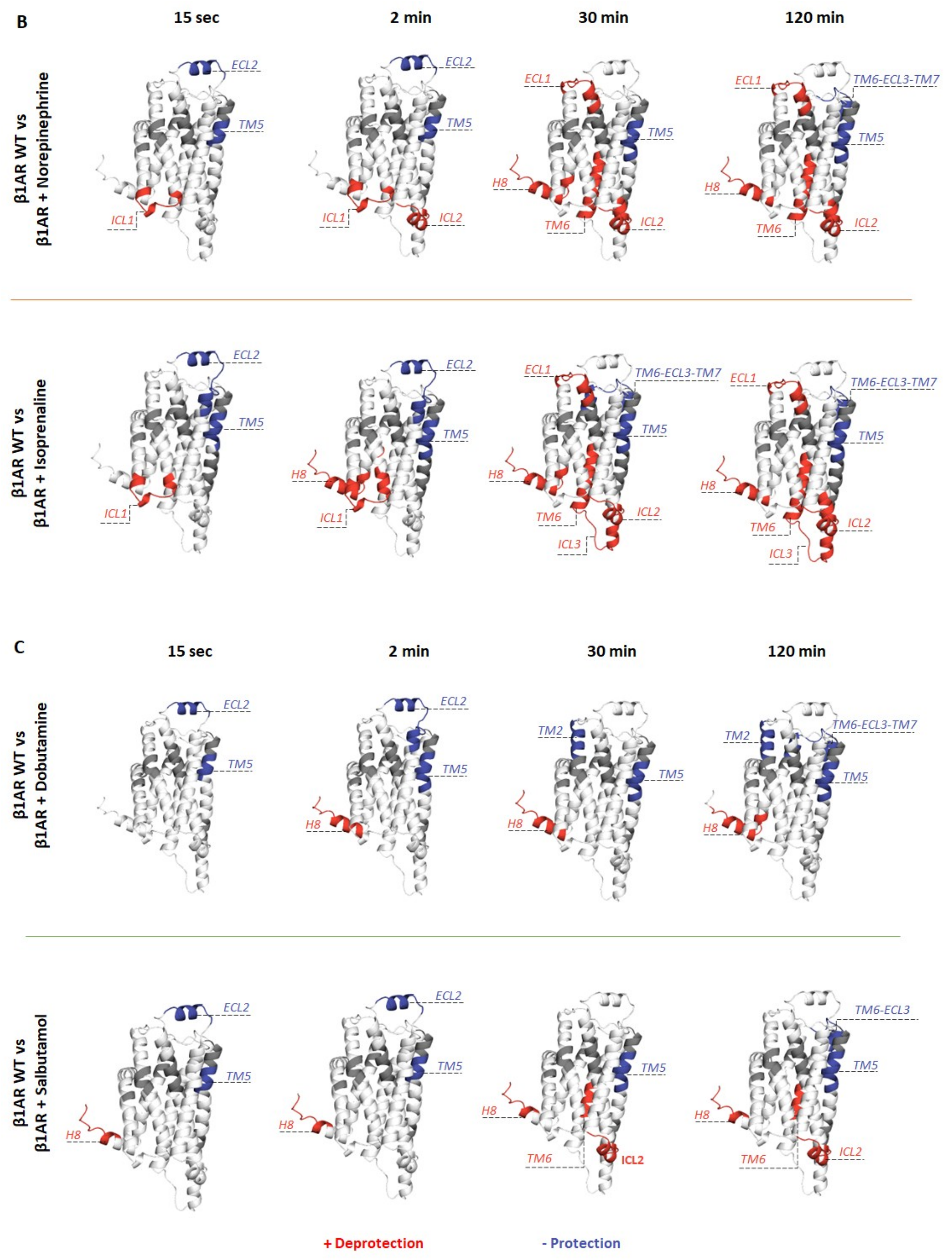

Supplementary Figure 12. HDX Experiment 1, 2 and 3. tβ1AR with ligands (antagonists, agonists and partial agonists). Graphical visualisation of the results obtained from the experiment 1,2 and 3. **A** Results mapped onto β1AR crystal structure (modelled by use of PDB structures; 2VT4 chain A and 6IBL chain A) for all tested antagonists. **B** Results for all tested agonists. **C** Results for all tested partial agonists. Differential deuterium uptake is plotted for each time point (15 sec, 2min, 30

min, 120 min). Red and blue indicate deprotection and protection respectively. Figures were created in PyMOL.

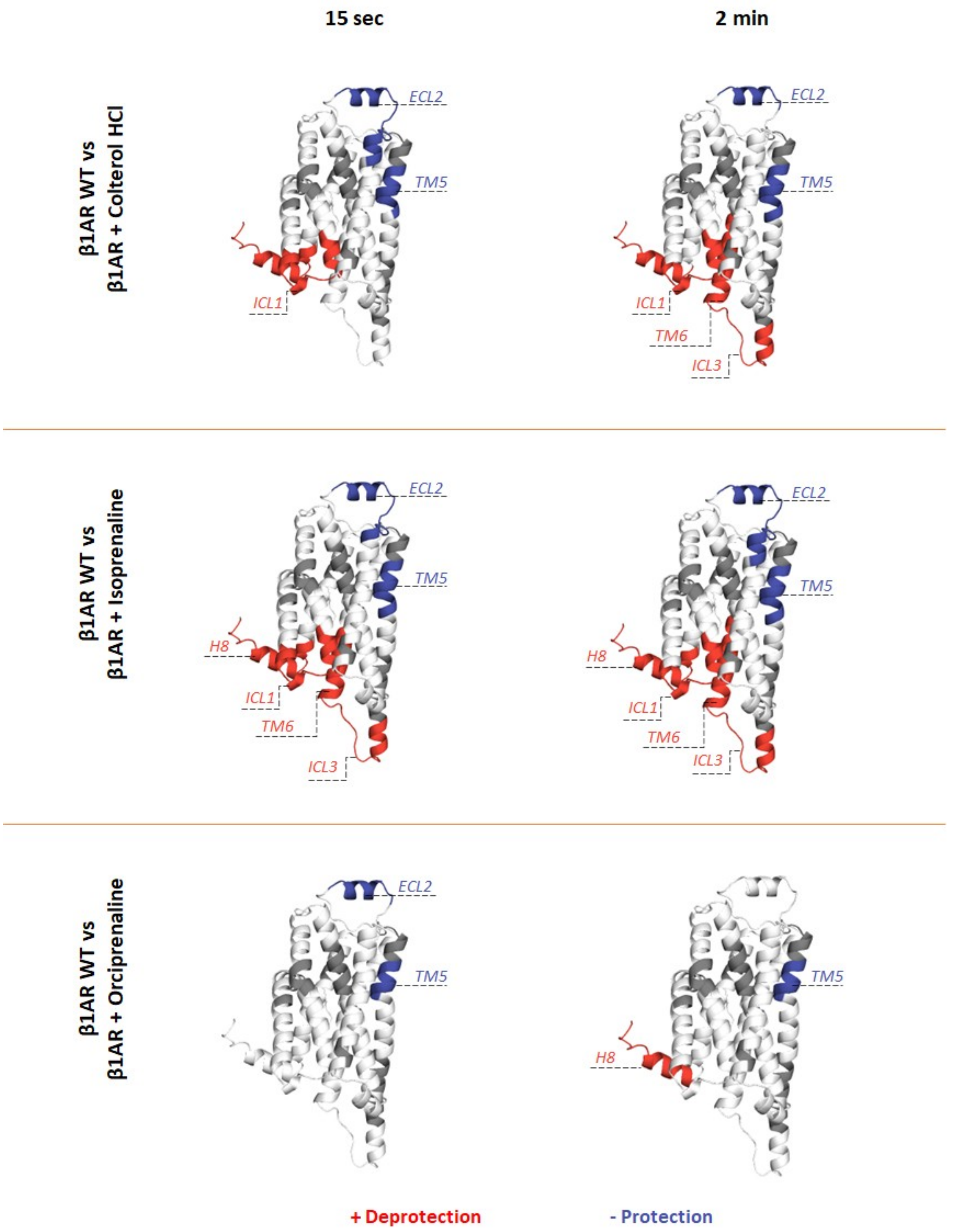

Supplementary Figure 13. HDX Experiment 4: tβ1AR with Isoprenaline derivatives. Graphical visualisation of the results obtained from the experiment 4. Observed effects for tβ1AR bound to Colterol HCl, Isoprenaline and Orciprenaline are mapped onto β1AR crystal structure (modelled by use of PDB structures; 2VT4 chain A and 6IBL chain A). Differential deuterium uptake is plotted for each time point (15 sec, 2min). Red and blue indicate deprotection and protection respectively. Figures were created in PyMOL.

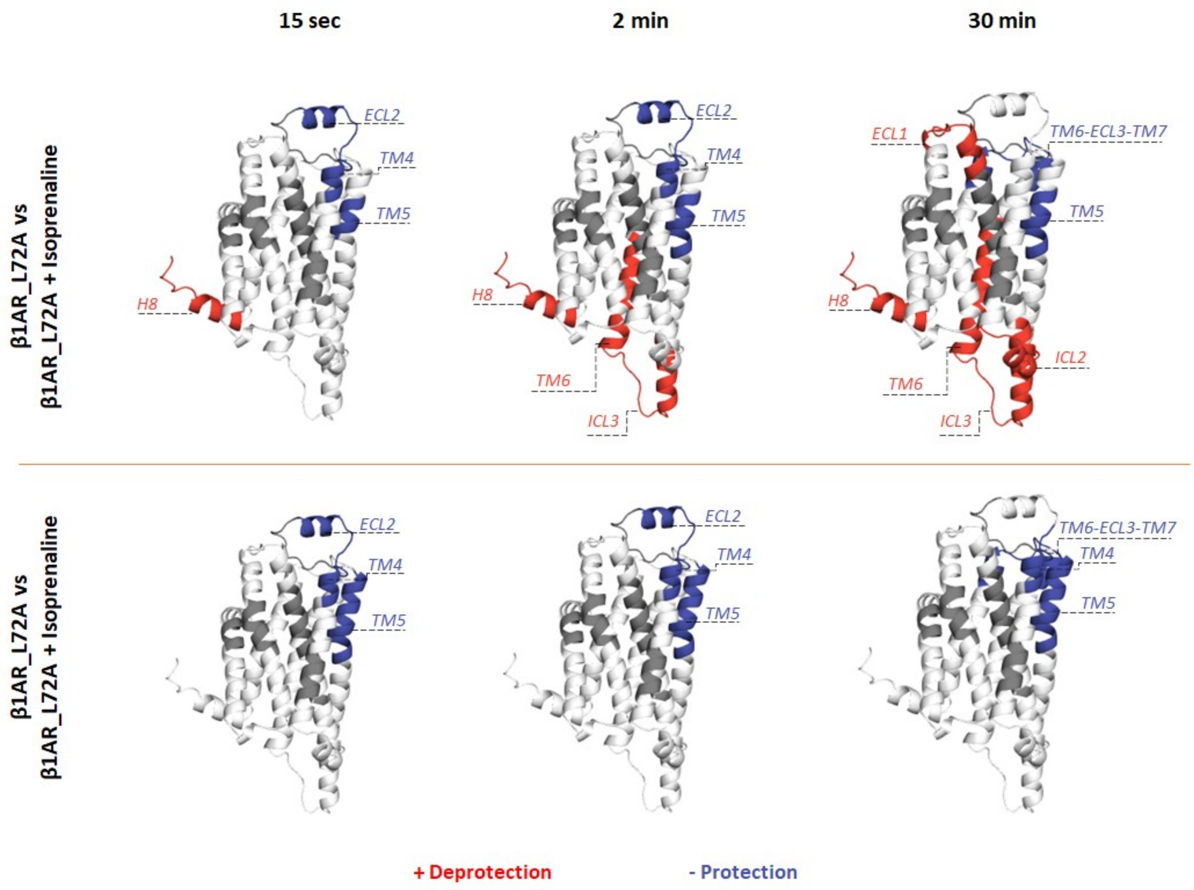

Supplementary Figure 14. HDX Experiment 5: tβ1AR_L72A mutant with Isoprenaline and Cyanopindolol. Graphical visualisation of the results obtained from the experiment 5. Observed effects for tβ1AR_L72A mutant bound to agonist isoprenaline and antagonist cyanopindolol are mapped onto β1AR crystal structure (modelled by use of PDB structures; 2VT4 chain A and 6IBL chain A). Differential deuterium uptake is plotted for each time point (15 sec, 2min, 30 min). Mutation of the residue L72 located on ICL1 abolished effects previously observed for that loop. Red and blue indicate deprotection and protection respectively. Figures were created in PyMOL.

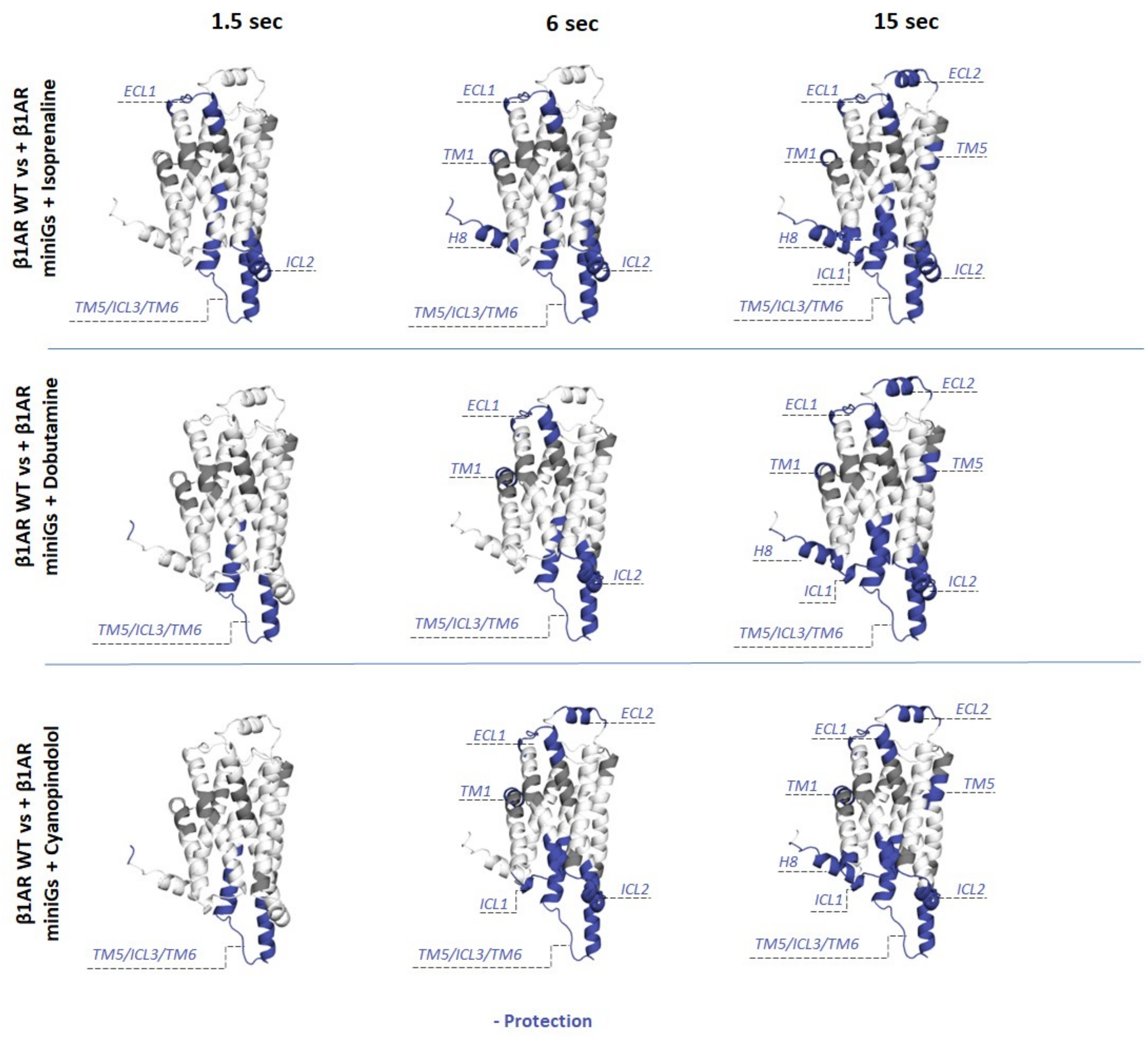
1.5 sec
6 sec
15 sec
β1AR WT vs + β1AR miniGs + Isoprenaline
β1AR WT vs + β1AR miniGs + Dobutamine
β1AR WT vs + β1AR miniGs + Cyanopindolol
ECL1
ECL2
TM1
TM5
H8
ICL1
ICL2
TM5/ICL3/TM6
- Protection

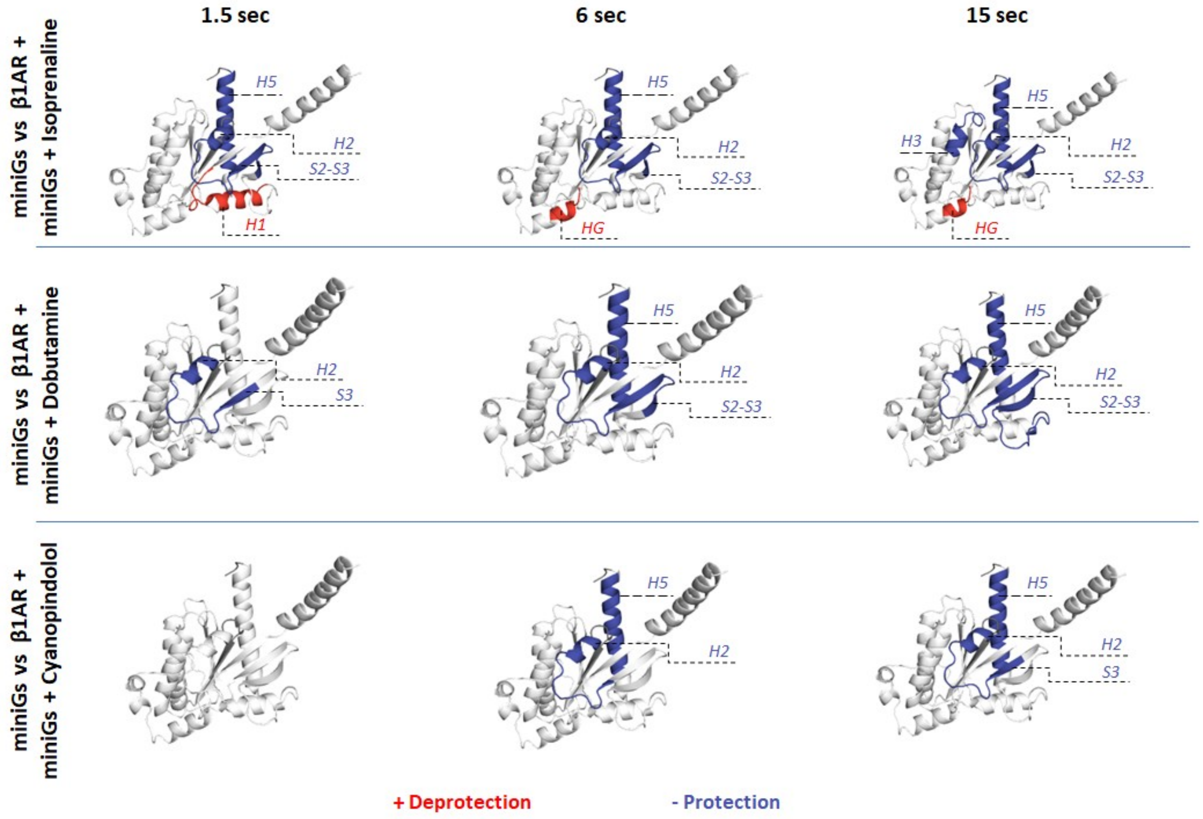

Supplementary Figure 15. HDX Experiment 6: tβ1AR in complex with miniGs and Isoprenaline, Dobutamine and Cyanopindolol. Graphical visualisation of the results obtained from the experiment 6. Observed effects for tβ1AR coupled to miniGs and agonist isoprenaline, antagonist cyanopindolol and partial agonist dobutamine, are mapped onto β1AR crystal structure (modelled by use of PDB structures; 2VT4 chain A and 6IBL chain A). Differential deuterium uptake is plotted for each time point (1.5 sec, 6 sec and 15 sec). Blue indicates protection and red deprotection. Figures were created in PyMOL.

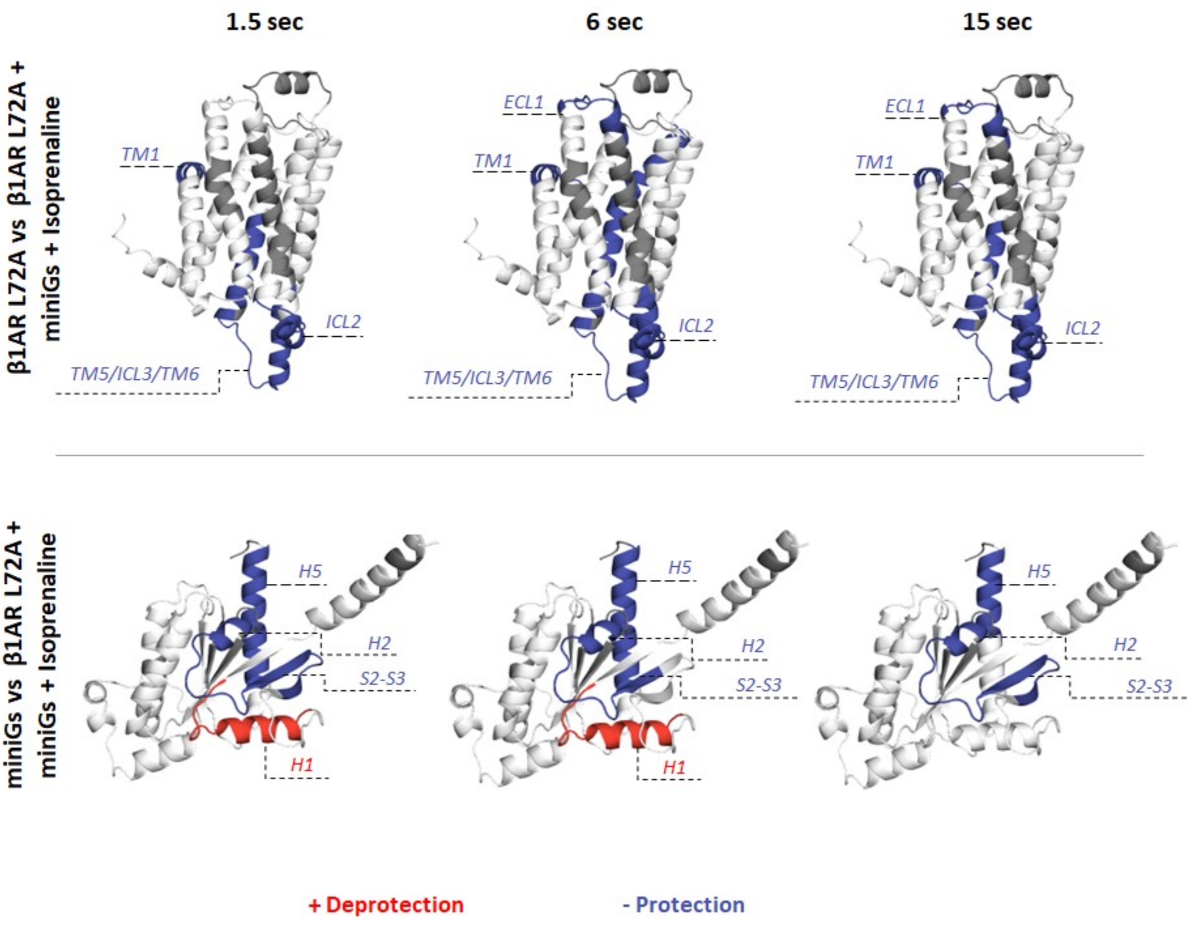

Supplementary Figure 16. HDX Experiment 7: tβ1AR L72A mutant in complex with miniGs and Isoprenaline. Graphical visualisation of the results obtained from the experiment 7. Observed effects for tβ1AR L72A mutant coupled to miniGs and agonist isoprenaline are mapped onto β1AR crystal structure (modelled by use of PDB structures; 2VT4 chain A and 6IBL chain A). Differential deuterium uptake is plotted for each time point (1.5 sec, 6 sec and 15 sec). Blue indicates protection and red deprotection. Figures were created in PyMOL.

Supplementary Figures 17-22

Uptake plots for all observed HDX effects for every experiment.

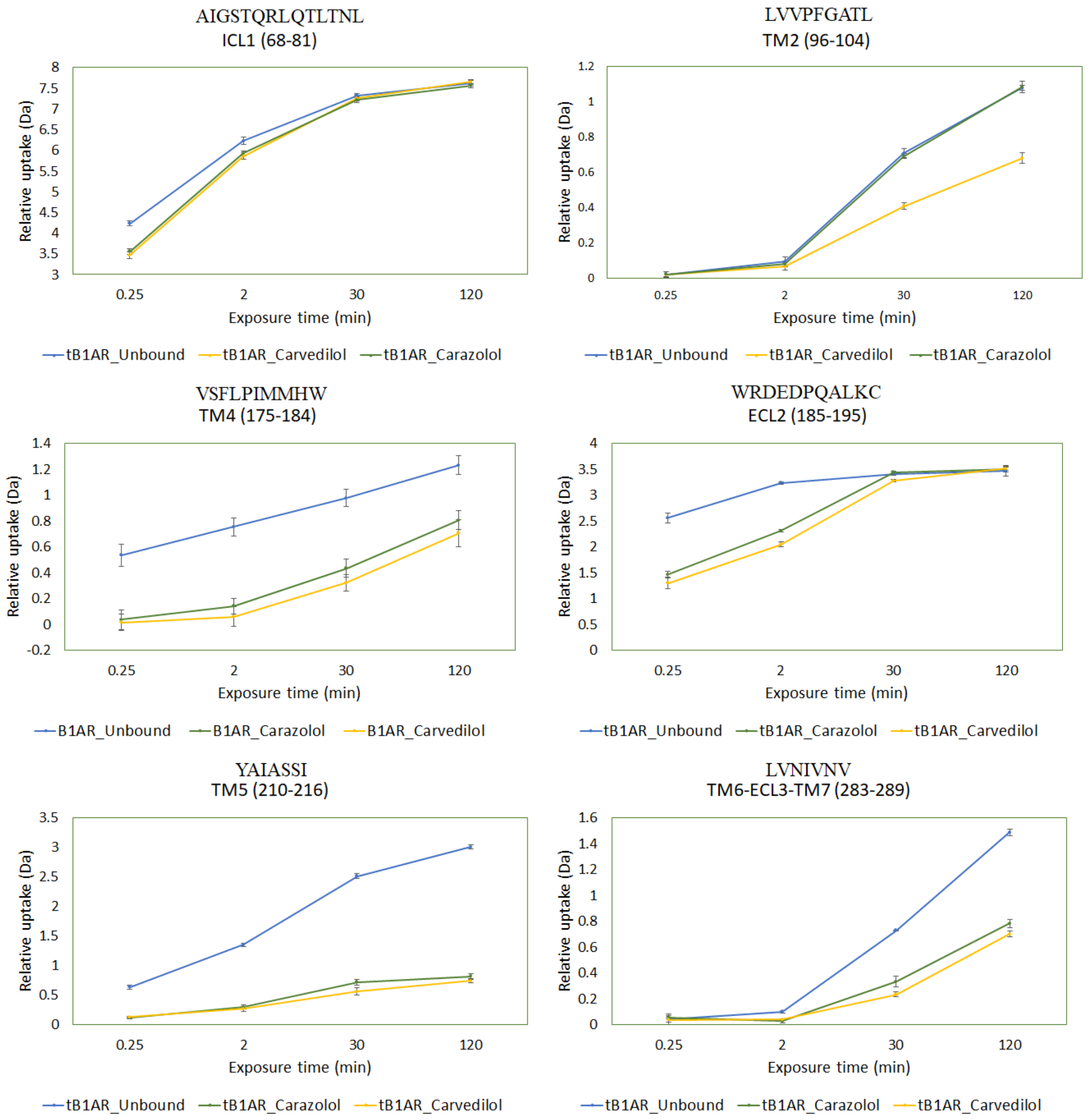

Supplementary Figure 17. Uptake plots for HDX experiment 1. The figure presents uptake plots of all observed effects for tβ1AR with antagonist Carazolol and Carvedilol. Data are presented as mean values ± SD, where error bars represent the standard deviation at each time point. Each measurement is based on three technical replicates (n = 3).

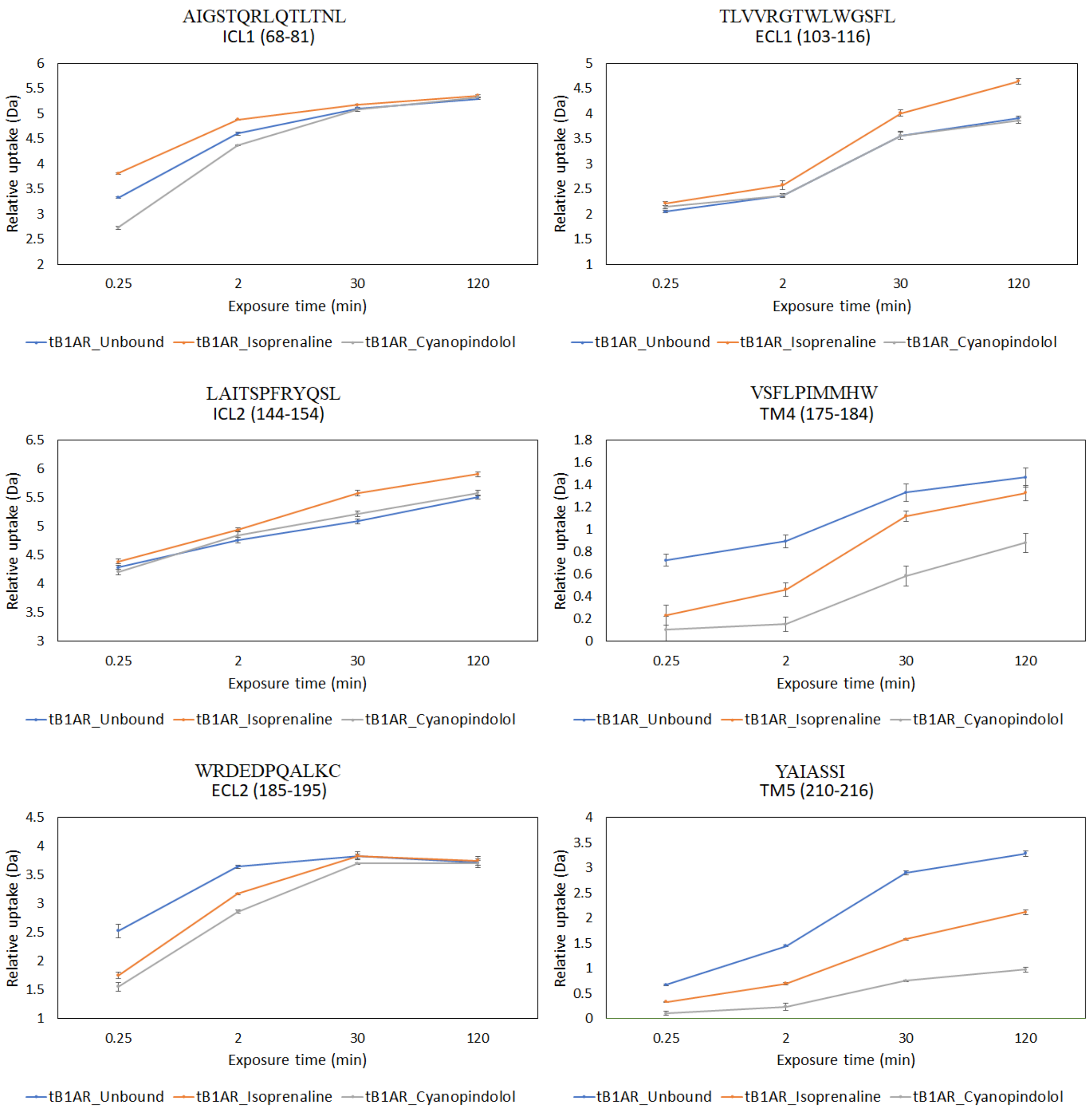
AIGSTQRLQTLTNL
ICL1 (68-81)
TLVVRGTWLWGSFL
ECL1 (103-116)
LAITSPFRYQSL
ICL2 (144-154)
VSFLPIMMHW
TM4 (175-184)
WRDEDPQALKC
ECL2 (185-195)
YAIASSI
TM5 (210-216)
Relative uptake (Da)
Exposure time (min)
0.25
2
30
120
tB1AR_Unbound
tB1AR_Isoprenaline
tB1AR_Cyanopindolol

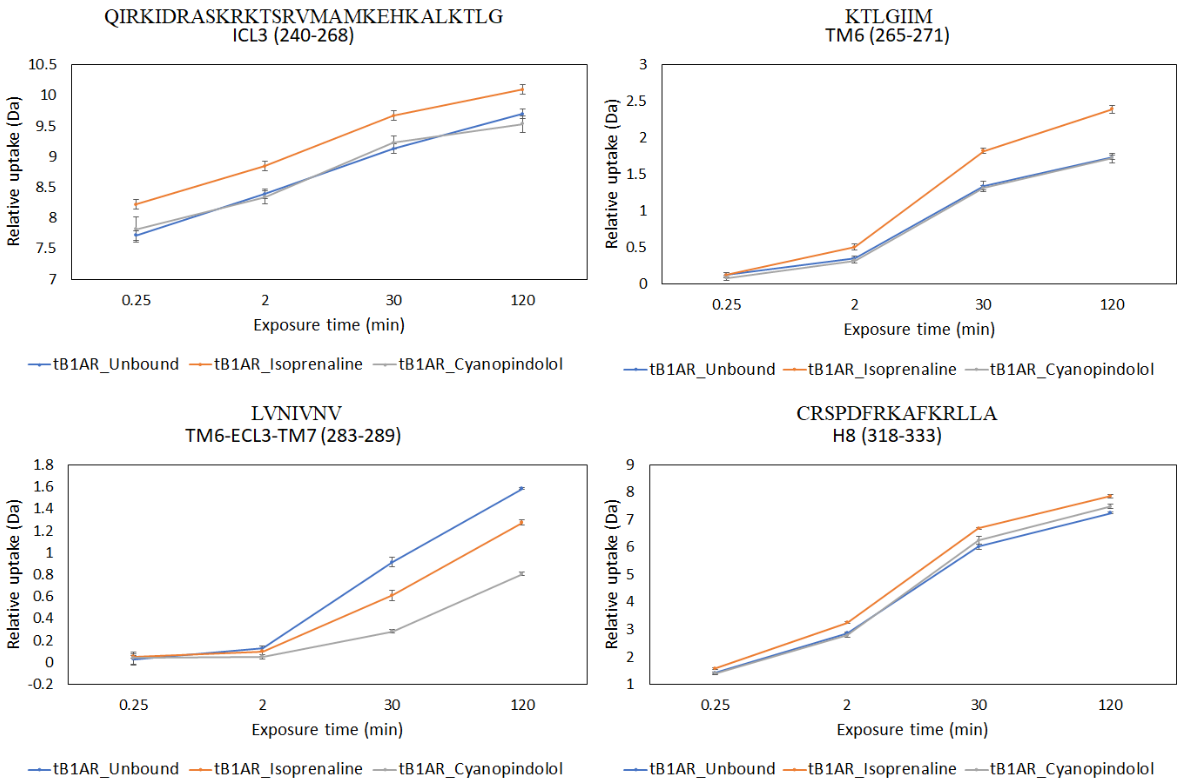

Supplementary Figure 18. Uptake plots for HDX experiment 2. The figure presents uptake plots of all observed effects for tβ1AR with antagonist Cyanopindolol and agonist Isoprenaline. Data are presented as mean values ± SD, where error bars represent the standard deviation at each time point. Each measurement is based on three technical replicates (n = 3).

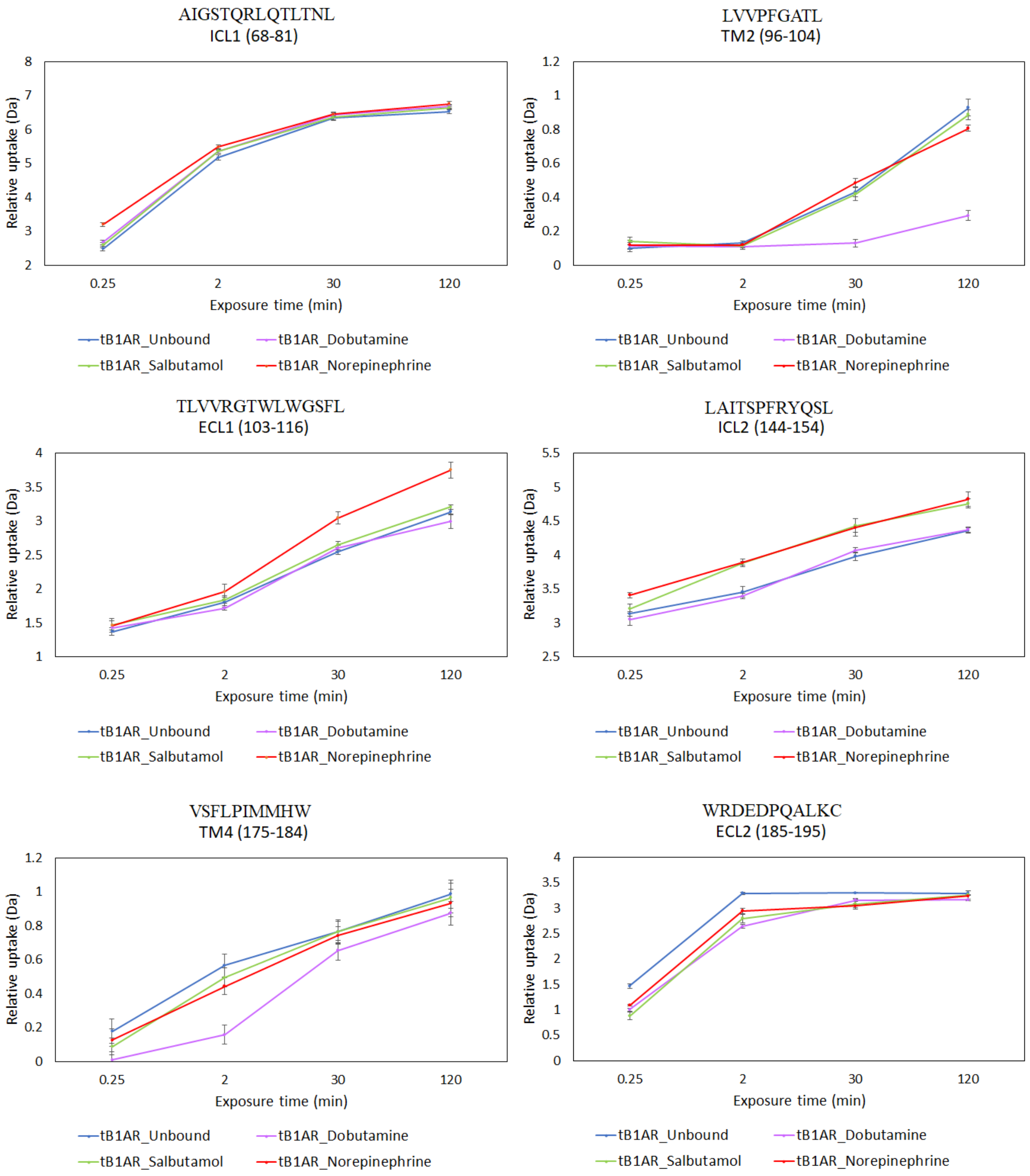
AIGSTQRLQTLTNL
ICL1 (68-81)
LVVPFGATL
TM2 (96-104)
TLVVRGTWLWGSFL
ECL1 (103-116)
LAITSPFRYQSL
ICL2 (144-154)
VSFLPIMMHW
TM4 (175-184)
WRDEDPQALKC
ECL2 (185-195)
Relative uptake (Da)
Exposure time (min)
0.25
2
30
120
tB1AR_Unbound
tB1AR_Dobutamine
tB1AR_Salbutamol
tB1AR_Norepinephrine

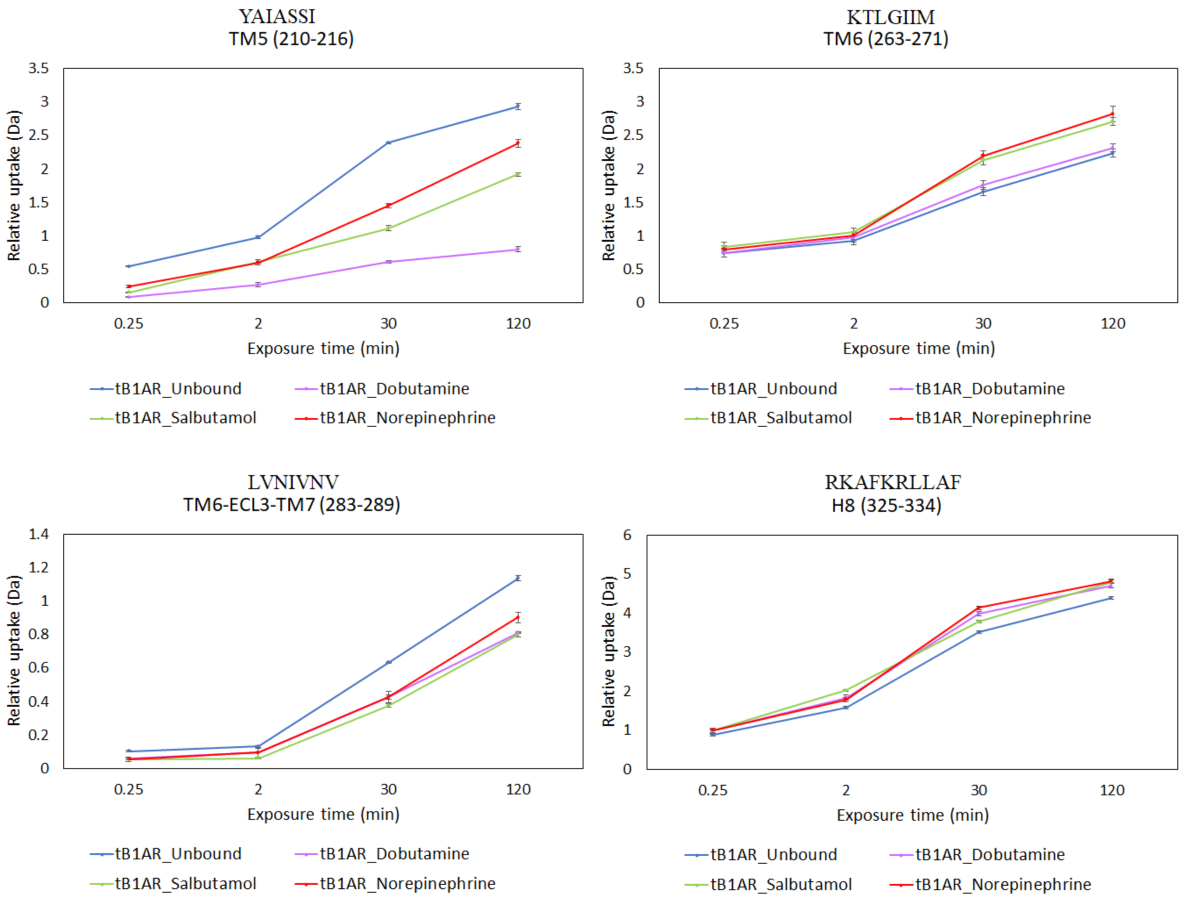

Supplementary Figure 19. Uptake plots for HDX experiment 3. The figure presents uptake plots of all observed effects for tβ1AR with agonist Norepinephrine and partial agonists Dobutamine and Salbutamol. Data are presented as mean values ± SD, where error bars represent the standard deviation at each time point. Each measurement is based on three technical replicates (n = 3).

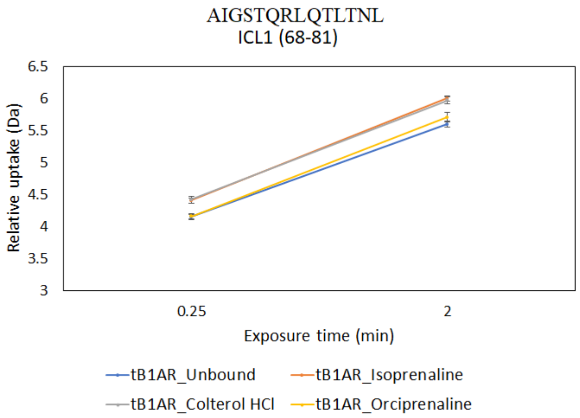
AIGSTQRLQTLTNL
ICL1 (68-81)
Relative uptake (Da)
6.5
6
5.5
5
4.5
4
3.5
3
0.25
2
Exposure time (min)
tB1AR_Unbound
tB1AR_Isoprenaline
tB1AR_Colterol HCl
tB1AR_Orciprenaline

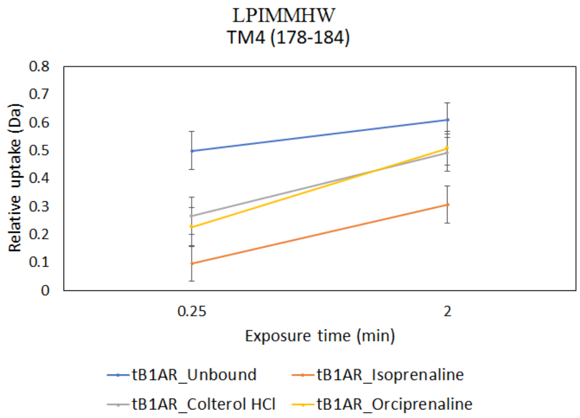
LPIMMHW
TM4 (178-184)
Relative uptake (Da)
0.8
0.7
0.6
0.5
0.4
0.3
0.2
0.1
0
0.25
2
Exposure time (min)
tB1AR_Unbound
tB1AR_Isoprenaline
tB1AR_Colterol HCl
tB1AR_Orciprenaline

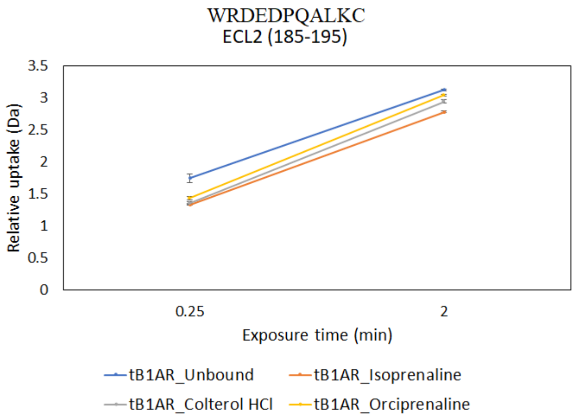
WRDEDPQALKC
ECL2 (185-195)
Relative uptake (Da)
3.5
3
2.5
2
1.5
1
0.5
0
0.25
2
Exposure time (min)
tB1AR_Unbound
tB1AR_Isoprenaline
tB1AR_Colterol HCl
tB1AR_Orciprenaline

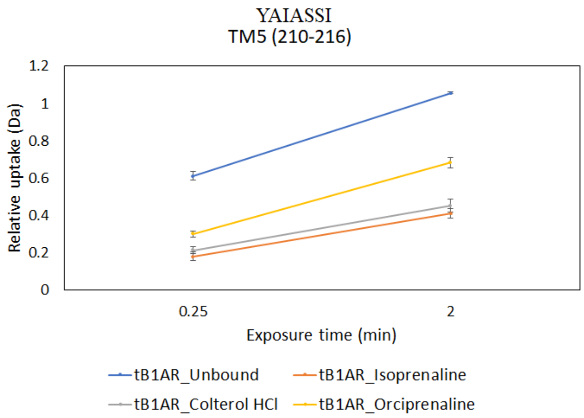
YAIASSI
TM5 (210-216)
Relative uptake (Da)
1.2
1
0.8
0.6
0.4
0.2
0
0.25
2
Exposure time (min)
tB1AR_Unbound
tB1AR_Isoprenaline
tB1AR_Colterol HCl
tB1AR_Orciprenaline

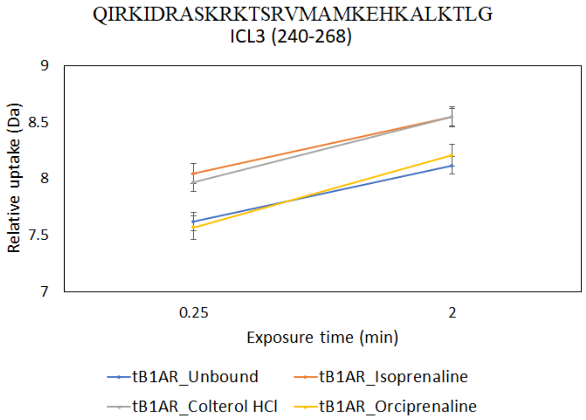
QIRKIDRASKRKTSRVMAMKEHKALKTLG
ICL3 (240-268)
Relative uptake (Da)
9
8.5
8
7.5
7
0.25
2
Exposure time (min)
tB1AR_Unbound
tB1AR_Isoprenaline
tB1AR_Colterol HCl
tB1AR_Orciprenaline

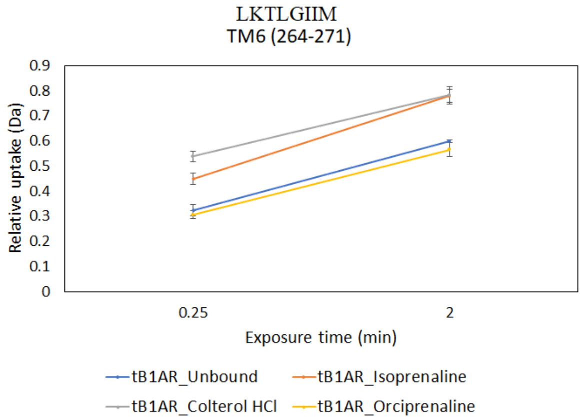
LKTLGIIM
TM6 (264-271)
Relative uptake (Da)
0.9
0.8
0.7
0.6
0.5
0.4
0.3
0.2
0.1
0
0.25
2
Exposure time (min)
tB1AR_Unbound
tB1AR_Isoprenaline
tB1AR_Colterol HCl
tB1AR_Orciprenaline

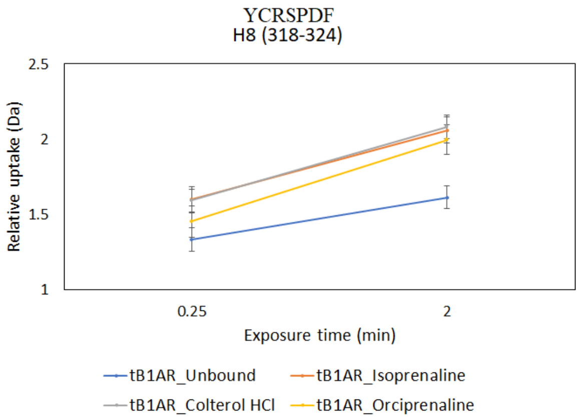
YCRSPDF
H8 (318-324)
Relative uptake (Da)
2.5
2
1.5
1
0.25
2
Exposure time (min)
tB1AR_Unbound
tB1AR_Isoprenaline
tB1AR_Colterol HCl
tB1AR_Orciprenaline

Supplementary Figure 20. Uptake plots for HDX experiment 4. The figure presents uptake plots of all observed effects for tβ1AR with Isoprenaline derivatives: Colterol HCl and Orciprenaline. Data are presented as mean values ± SD, where error bars represent the standard deviation at each time point. Each measurement is based on three technical replicates (n = 3).

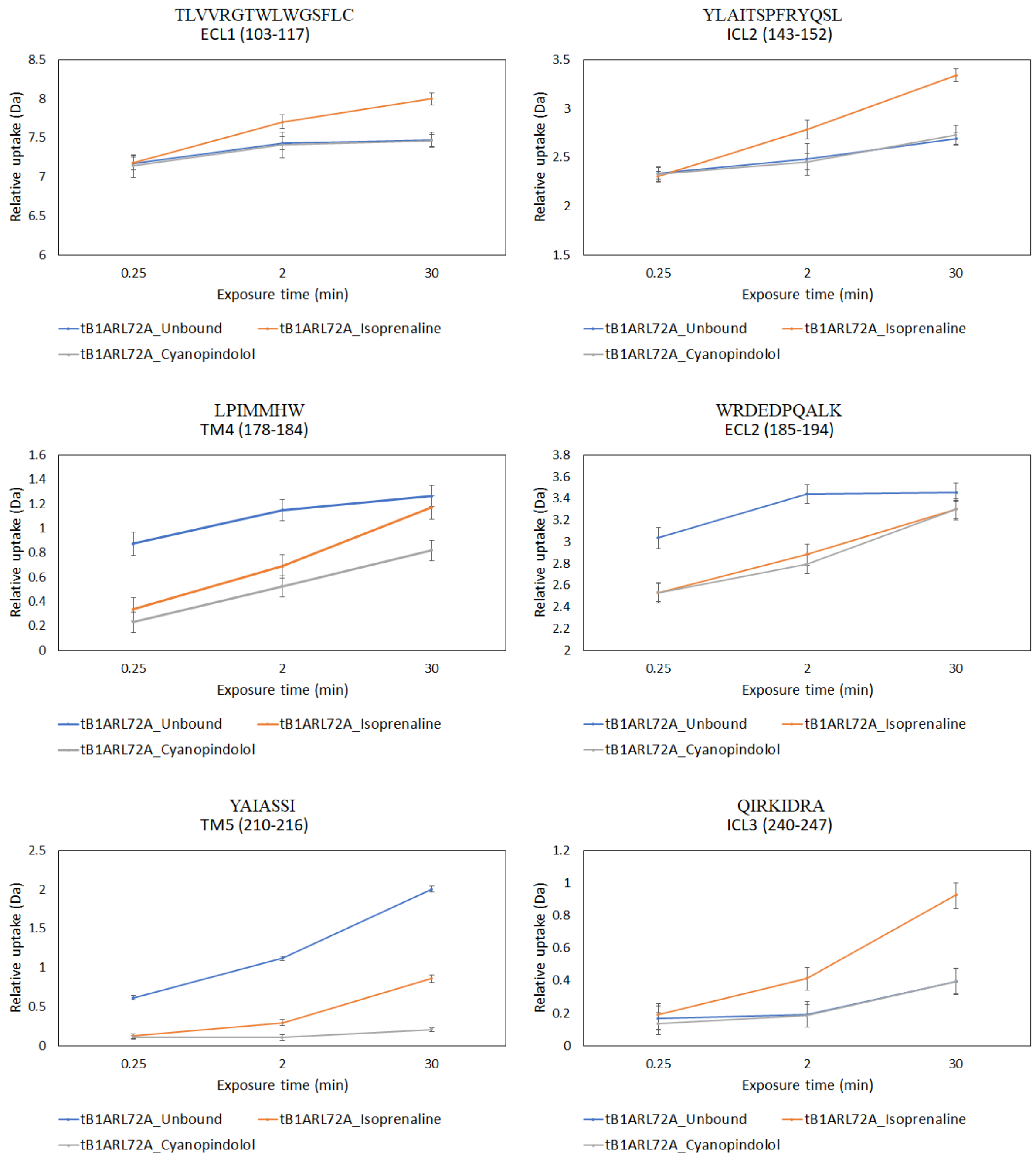

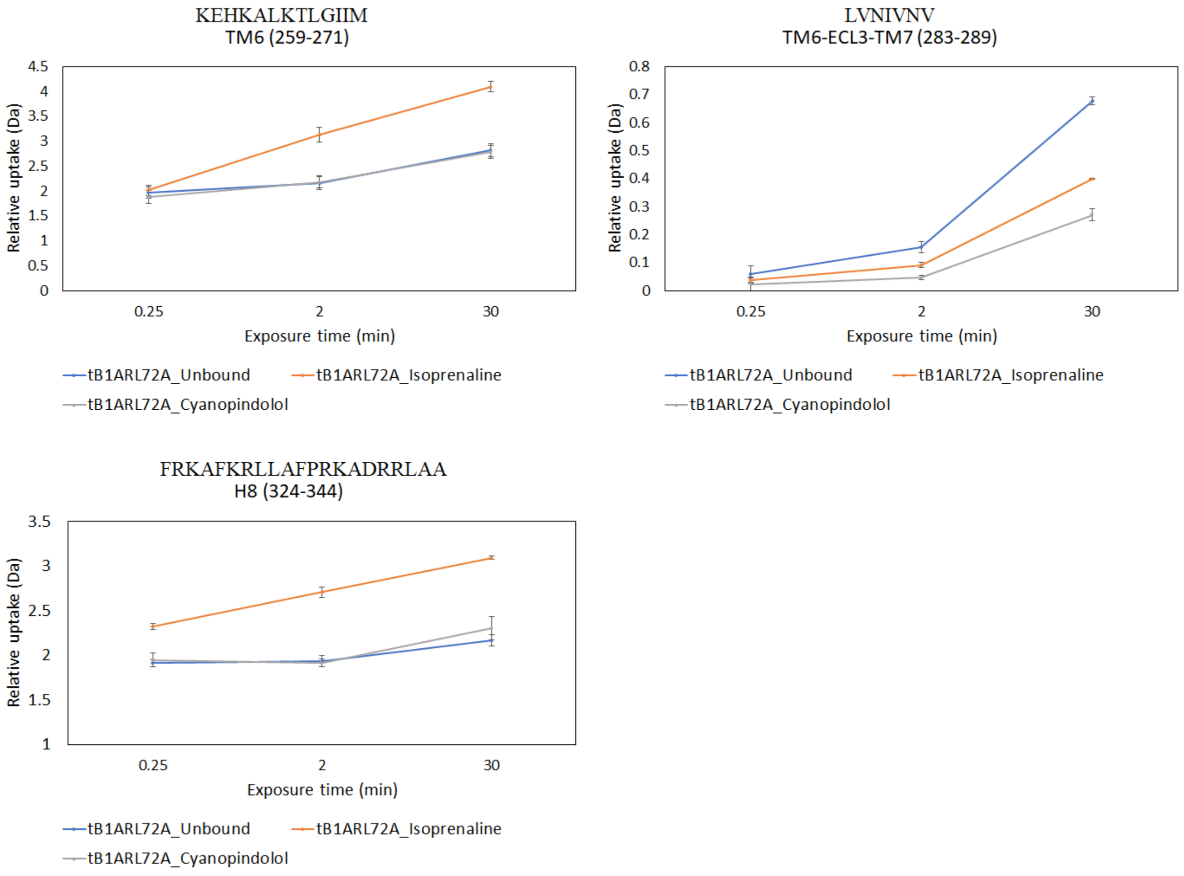

Supplementary Figure 21. Uptake plots for HDX experiment 5. The figure presents uptake plots of all observed effects for tβ1ARL72A mutant with agonist Isoprenaline and antagonist Cyanopindolol. Data are presented as mean values ± SD, where error bars represent the standard deviation at each time point. Each measurement is based on three technical replicates (n = 3).

A

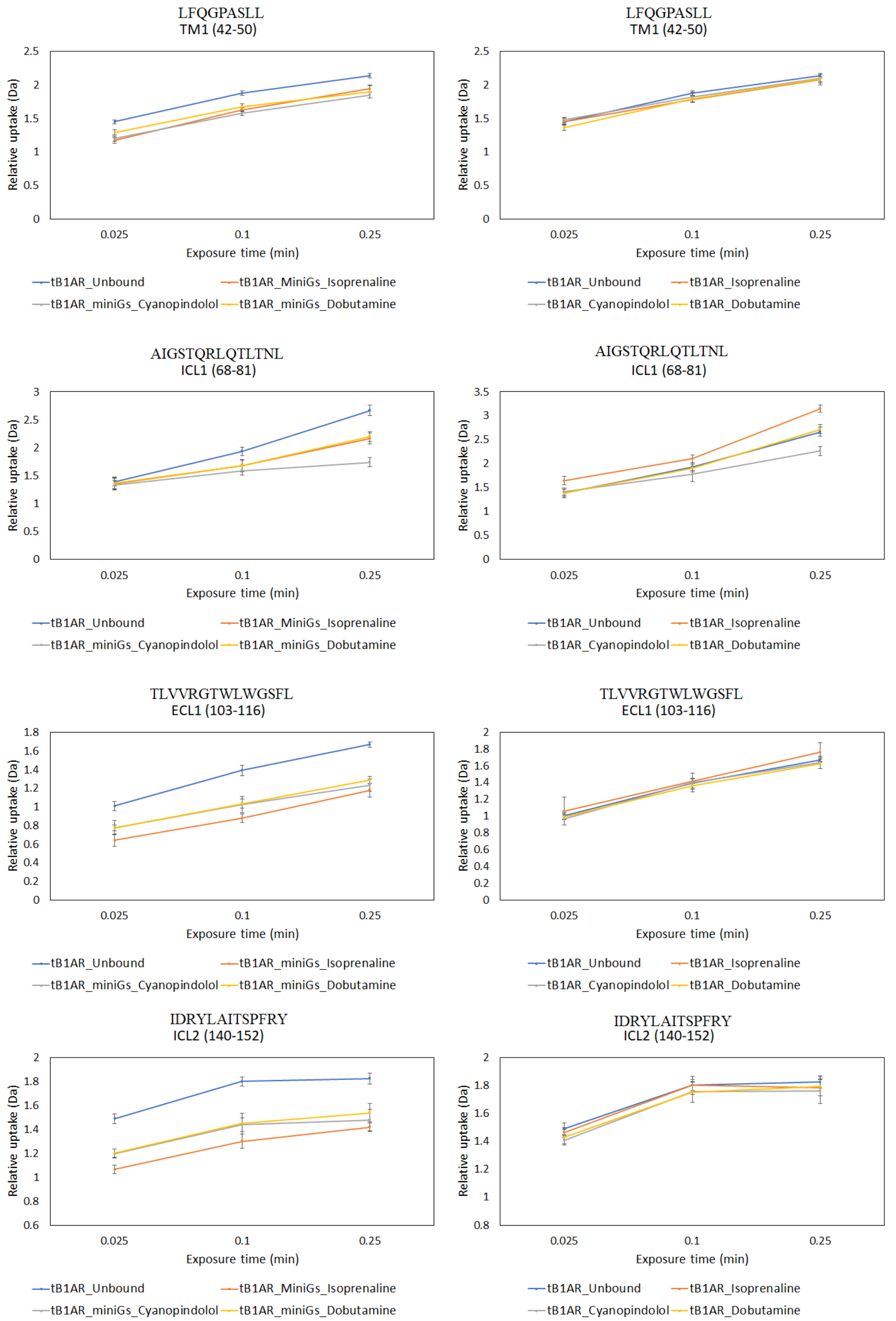
LFQGPASLL
TM1 (42-50)
Relative uptake (Da)
Exposure time (min)
0.025
0.1
0.25
tB1AR_Unbound
tB1AR_MiniGs_Isoprenaline
tB1AR_miniGs_Cyanopindolol
tB1AR_miniGs_Dobutamine
LFQGPASLL
TM1 (42-50)
tB1AR_Unbound
tB1AR_Isoprenaline
tB1AR_Cyanopindolol
tB1AR_Dobutamine
AIGSTQRLQTLTNL
ICL1 (68-81)
AIGSTQRLQTLTNL
ICL1 (68-81)
TLVVRGTWLWGSFL
ECL1 (103-116)
tB1AR_miniGs_Isoprenaline
TLVVRGTWLWGSFL
ECL1 (103-116)
IDRYLAITSPFRY
ICL2 (140-152)
IDRYLAITSPFRY
ICL2 (140-152)

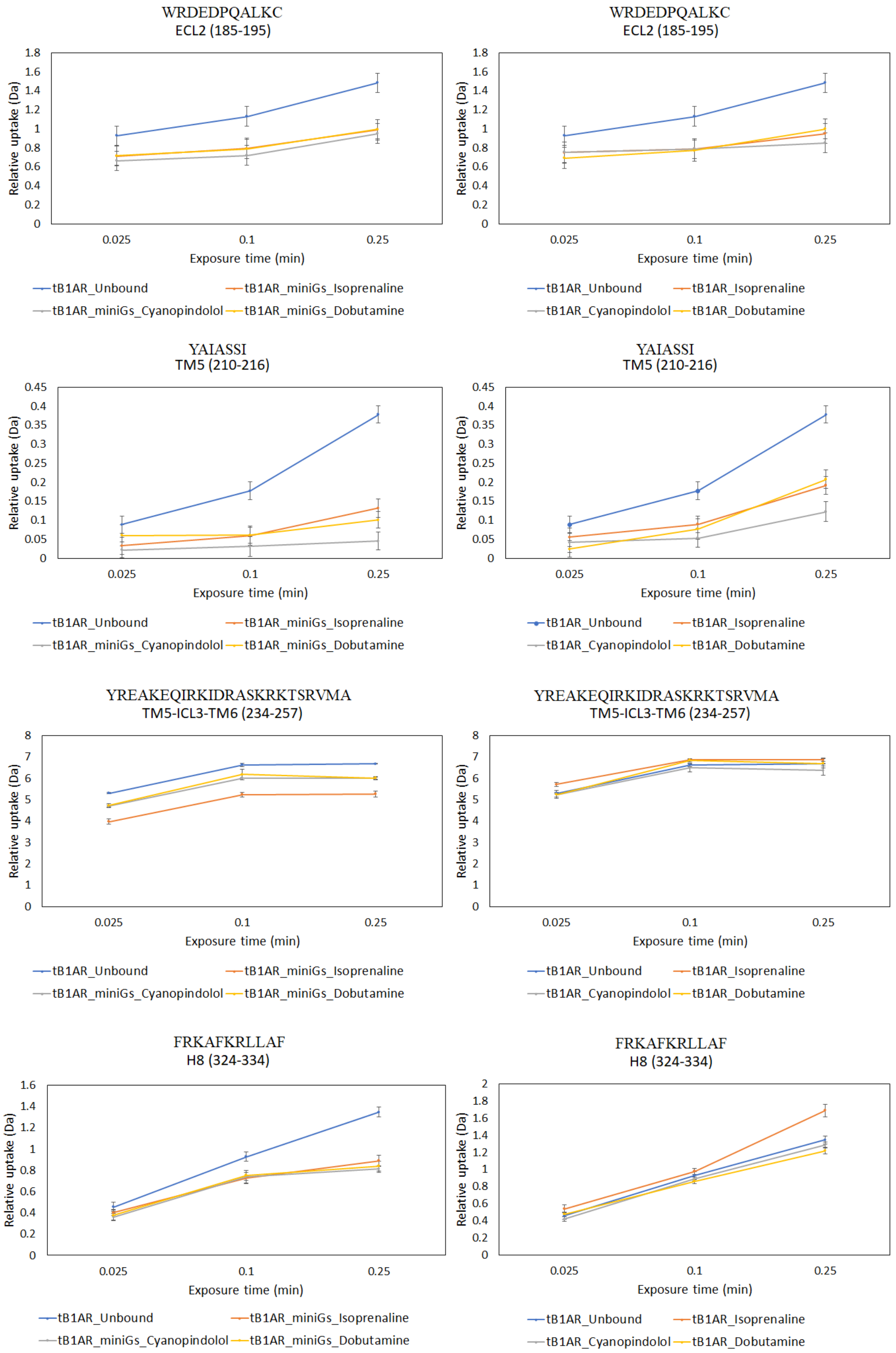

WRDEDPQALKC
ECL2 (185-195)
Relative uptake (Da)
Exposure time (min)
0.025
0.1
0.25
tB1AR_Unbound
tB1AR_miniGs_Isoprenaline
tB1AR_miniGs_Cyanopindolol
tB1AR_miniGs_Dobutamine
WRDEDPQALKC
ECL2 (185-195)
Relative uptake (Da)
Exposure time (min)
tB1AR_Unbound
tB1AR_Isoprenaline
tB1AR_Cyanopindolol
tB1AR_Dobutamine
YAIASSI
TM5 (210-216)
Relative uptake (Da)
Exposure time (min)
tB1AR_Unbound
tB1AR_miniGs_Isoprenaline
tB1AR_miniGs_Cyanopindolol
tB1AR_miniGs_Dobutamine
YAIASSI
TM5 (210-216)
Relative uptake (Da)
Exposure time (min)
tB1AR_Unbound
tB1AR_Isoprenaline
tB1AR_Cyanopindolol
tB1AR_Dobutamine
YREAKEQIRKIDRASKRKTSRVMA
TM5-ICL3-TM6 (234-257)
Relative uptake (Da)
Exposure time (min)
tB1AR_Unbound
tB1AR_miniGs_Isoprenaline
tB1AR_miniGs_Cyanopindolol
tB1AR_miniGs_Dobutamine
YREAKEQIRKIDRASKRKTSRVMA
TM5-ICL3-TM6 (234-257)
Relative uptake (Da)
Exposure time (min)
tB1AR_Unbound
tB1AR_Isoprenaline
tB1AR_Cyanopindolol
tB1AR_Dobutamine
FRKAFKRLLAF
H8 (324-334)
Relative uptake (Da)
Exposure time (min)
tB1AR_Unbound
tB1AR_miniGs_Isoprenaline
tB1AR_miniGs_Cyanopindolol
tB1AR_miniGs_Dobutamine
FRKAFKRLLAF
H8 (324-334)
Relative uptake (Da)
Exposure time (min)
tB1AR_Unbound
tB1AR_Isoprenaline
tB1AR_Cyanopindolol
tB1AR_Dobutamine

B

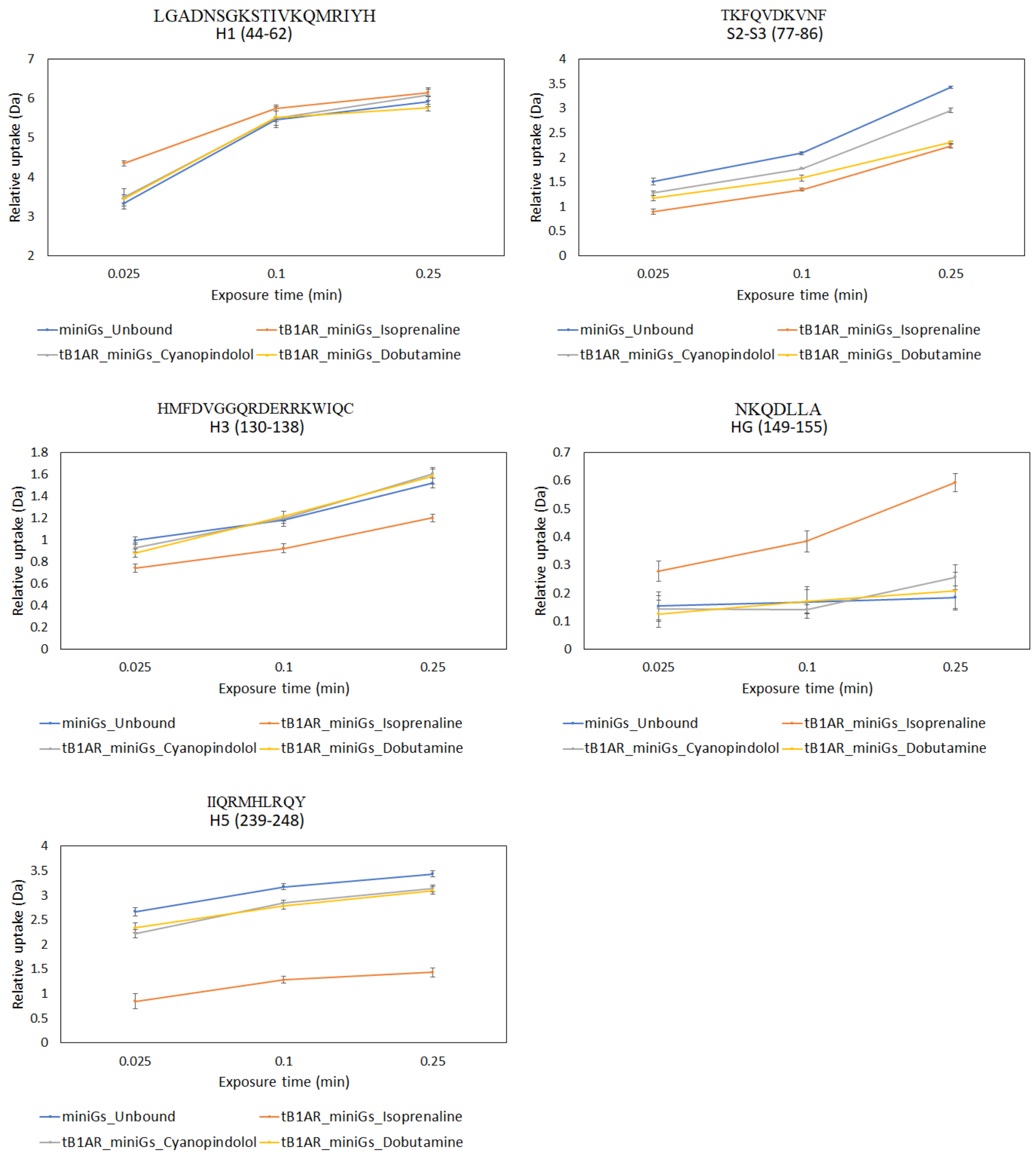

Supplementary Figure 21. Uptake plots for HDX experiment 6. The figure presents uptake plots of all observed effects for tβ1AR in complex with miniGs and agonist Isoprenaline, partial agonist Dobutamine and antagonist Cyanopindolol. **A** Uptake plots of the effects observed on the regions of tβ1AR, where he plots on the right side depict control runs performed simultaneously without miniGs. **B** Uptake plots of the effects observed on the regions of miniGs. Data are presented as mean values ± SD, where error bars represent the standard deviation at each time point. Each measurement is based on three technical replicates (n = 3).

A

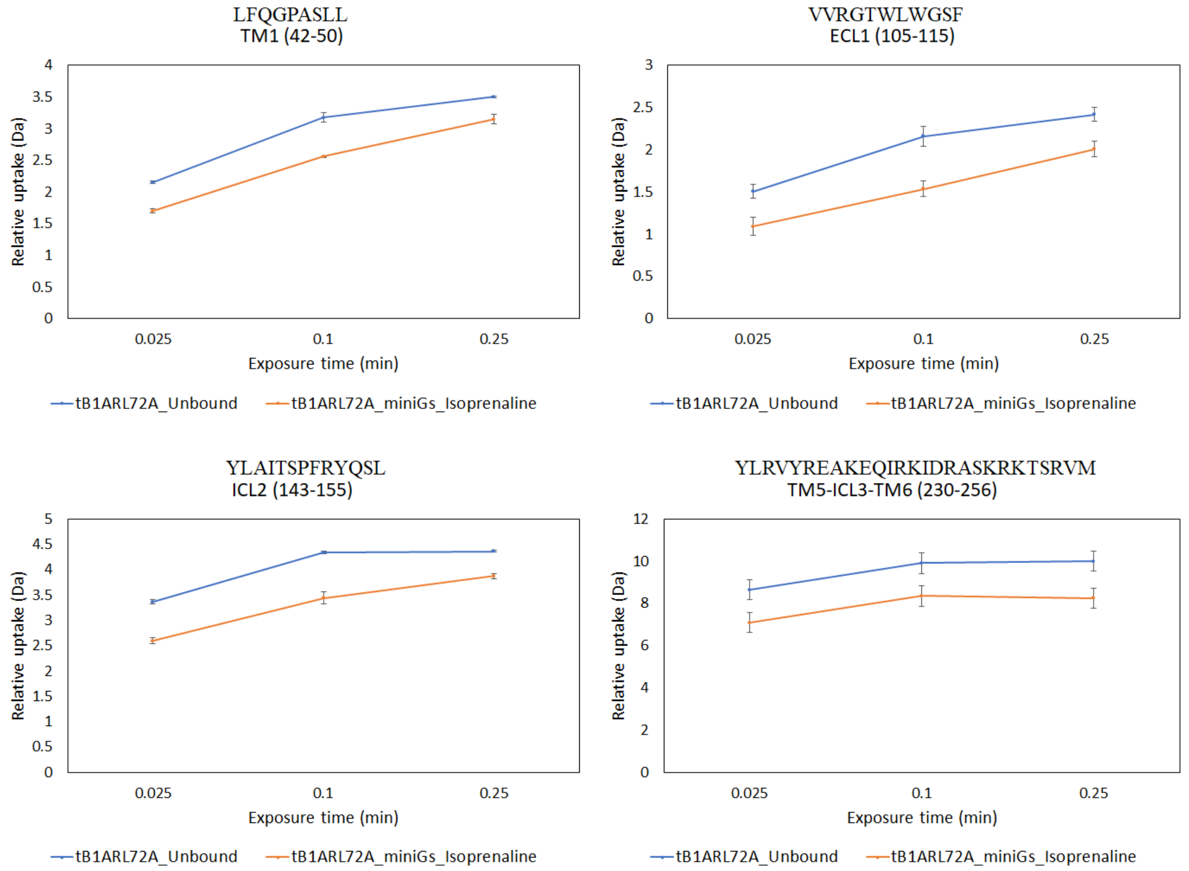
LFQGPASLL
TM1 (42-50)
Relative uptake (Da)
4
3.5
3
2.5
2
1.5
1
0.5
0
0.025
0.1
0.25
Exposure time (min)
tB1ARL72A_Unbound
tB1ARL72A_miniGs_Isoprenaline
VVRGTWLWGSF
ECL1 (105-115)
Relative uptake (Da)
3
2.5
2
1.5
1
0.5
0
0.025
0.1
0.25
Exposure time (min)
tB1ARL72A_Unbound
tB1ARL72A_miniGs_Isoprenaline
YLAITSPFRYQSL
ICL2 (143-155)
Relative uptake (Da)
5
4.5
4
3.5
3
2.5
2
1.5
1
0.5
0
0.025
0.1
0.25
Exposure time (min)
tB1ARL72A_Unbound
tB1ARL72A_miniGs_Isoprenaline
YLRVYREAKEQIRKIDRASKRKTSRVM
TM5-ICL3-TM6 (230-256)
Relative uptake (Da)
12
10
8
6
4
2
0
0.025
0.1
0.25
Exposure time (min)
tB1ARL72A_Unbound
tB1ARL72A_miniGs_Isoprenaline

B

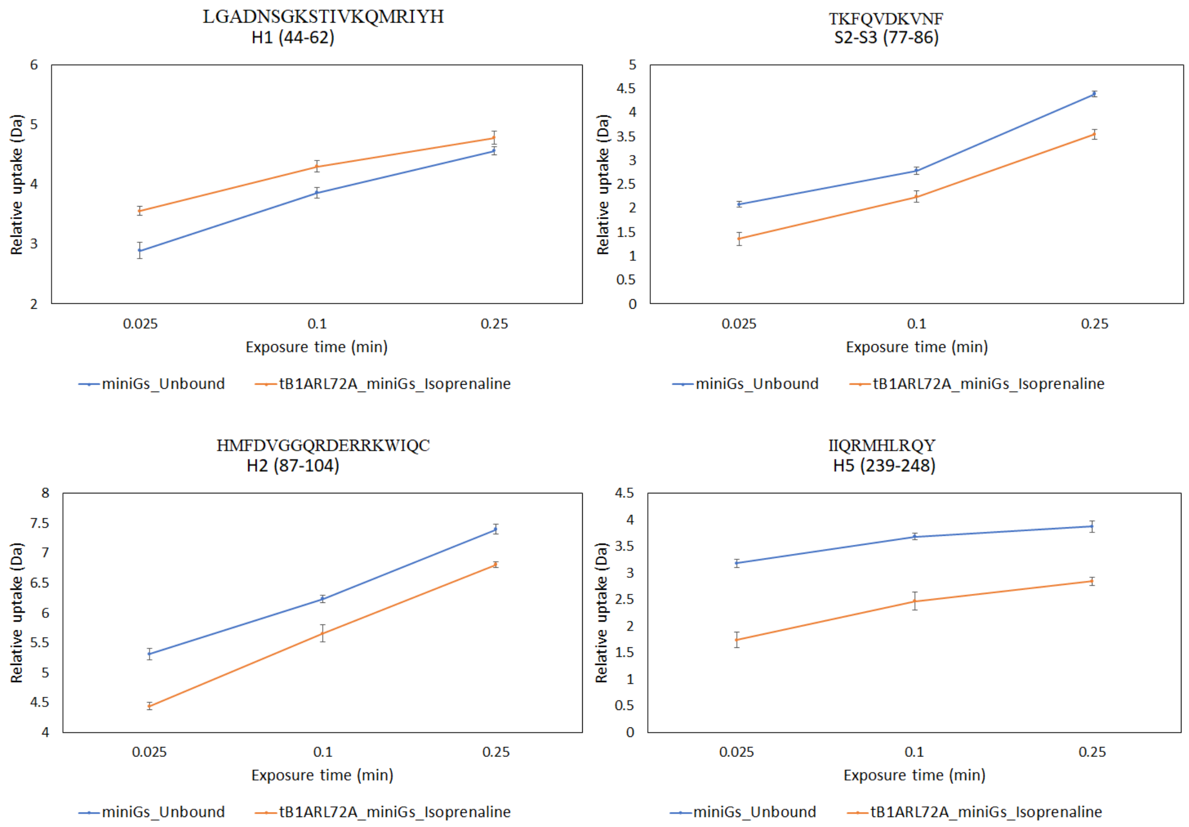

Supplementary Figure 22. Uptake plots for HDX experiment 7. The figure presents uptake plots of all observed effects for tβ1ARL72A mutant in complex with miniGs and agonist Isoprenaline. **A** Uptake plots of the effects observed on the regions of tβ1AR. **B** Uptake plots of the effects observed on the regions of miniGs. Data are presented as mean values ± SD, where error bars represent the standard deviation at each time point. Each measurement is based on three technical replicates (n = 3).

3. Supplementary Tables.

A

Quench composition	Digestion coverage	Number of peptides	Redundancy
Waters pepsin + BPR+ 0.02% DDM	30.4%	18	1.60
Waters pepsin + 0.02% DDM	30.1%	17	1.47
Waters pepsin + 0.02% DDM, SD	16.6%	7	1.17
Waters pepsin + 0.1%DDM	50%	29	1.62
Waters pepsin + 0.02% DDM + 6M Gnd-HCl	42.8%	22	1.48
Waters pepsin + 0.02% DDM + 6M urea	43.1%	23	1.63
Waters pepsin + 0.1% DDM (8 to 55% LC)	44.5%	31	1.93
Waters pepsin + 0.1% DDM + 400mM TCEP	42%	27	1.78

B

Quench composition	Digestion coverage	Number of peptides	Redundancy
DIY PC + 0.1%DDM	75.4%	93	3.68
DIY PC + CELUT + 0.1%DDM	86.2%	108	3.78
DIY PC + 0.1%DDM, CE	85%	111	4.01
DIY PC + 0.1%DDM, CE, SD	85.5%	112	4.06
DIY PC+ 0.1%DDM, 15mM TCEP, CE	74%	72	3.18
DIY PC + 0.1%DDM, 50mM TCEP, CELUT	90.1%	125	4.27
DIY PC + 0.1% DDM, 100mM TCEP, CELUT	90.1%	142	4.97
DIY PC+ 0.1%DDM_400mM TCEP, CELUT	61%	50	2.43*
DIY + 0.1%DDM, Rhizopus	93.4%	130	4.80
DIY PC+0.1%DDM, 100mM TCEP, C8	85.1%	120	4.31
DIY PC + 0.1%DDM, 4M Gnd-HCl	32%	18	1.48
DIY PC + 0.1%DDM, 6M Urea	66%	55	2.50
Dual Protease + 0.1% DDM, 100mM TCEP, CELUT	94%	263	8.37

Supplementary table 1. Results, as measured by digestion coverage, number of peptides identified, and redundancy obtained from DynamX for tβ1AR quench buffer optimisation using the Waters EnzymateTH BEH pepsin column (**A**), self-packed pepsin column and dual protease type XIII (**B**). Orange highlight indicates final conditions, that have been applied to every HDX experiment. Abbreviations: BPR – back pressure regulator, SD – slow digestion, Gnd-HCL – guanidine

hydrochloride, PC – pepsin column, DIY PC – self-packed pepsin column, CE – collision energy, CELUT – look up table collision energy, C8 – analytical column C8.

Ligands	Kd [uM]	Ligand Concentration [uM]	Protein Concentration [uM]	% P bound
Isoprenaline	0.870	300	11	99.70%
Norepinephrine	1.819	300	11	99.37%
Dobutamine	5.88	1000	11	99.41%
Salbutamol	20.89	1820	11	98.86%
Cyanopindolol	0.0000407	300	11	100%
Carvedilol	0.004	300	11	100%
Carazolol	0.000204	300	11	100%

Supplementary table 2. Table displaying the properties of the ligands and protein used for experiments. The summary of the ligands Kd calculated based on the logKd values published by Jillian G Baker[2]. Ligand concentration was calculated in order to maximise the % of the ligand bound to the receptor in the labelling experiment. It consists of basic Kd calculations including the dilution factor, which in this case was equal to 9. Almost all ligands have high affinity to the receptor, apart from dobutamine and salbutamol. Therefore, concentrations of those ligands were significantly higher than others. Only salbutamol has slightly below 99% receptor occupancy due to its low solubility in DMSO and high Kd.

	Antagonist			Agonist		Partial agonist	
**Motifs**	**Cyanopindolol**	**Carazolol**	**Carvedilol**	**Isoprenaline**	**Norepinephrine**	**Dobutamine**	**Salbutamol**
ECL1				+	+		
TM2			−			−	
TM5	−	−	−	−	−	−	−
TM6-ECL3-TM7	−	−	−	−	−	−	−
ECL2	−	−	−	−	−	−	−
TM4	−	−	−	−		−	
ICL1	−	−	−	+	+		
ICL2				+	+		+
ICL3				+			
TM6				+	+		+
H8				+	+	+	+

Supplementary table 3. Summary table of all observed effects for tβ1AR in complex with various ligands. Table represents tβ1AR motifs where significant differences in deuterium uptake were observed, between tβ1AR – apo state and tβ1AR – ligand state. Blue coloured columns indicate antagonists, orange – agonists and green – partial agonists. Minus indicates protection from exchange and plus indicates deprotection.

Supplementary Tables 4 – 10

HDX data tables recommended by community[3].

	tβ1AR apo + Carazolol	*tβ1AR apo*
**HDX reaction details**	50 mM K_2HPO_4 + 50 mM KH_2PO_4 + 100 mM TCEP + 0.1% DDM	
**HDX time course (min)**	0.25, 2, 30, 120 minutes	
**Number of peptides**	201	201
**Sequence coverage**	90.06%	90.06%
**Average peptide length/redundancy**	10.87 / 6.70	10.87 / 6.70
**Replicates (technical)**	3	3
**Repeatability (average SD)**	0.0611	0.0660
Significant difference in sum ΔHDX	CI 99% ± 0.34 Da	

	tβ1AR apo + Carvedilol	*tβ1AR apo*
**HDX reaction details**	50 mM K_2HPO_4 + 50 mM KH_2PO_4 + 100 mM TCEP + 0.1% DDM	
**HDX time course (min)**	0.25, 2, 30, 120 minutes	
**Number of peptides**	201	201
**Sequence coverage**	90.06%	90.06%
**Average peptide length/redundancy**	10.87 / 6.70	10.87 / 6.70
**Replicates (technical)**	3	3
**Repeatability (average SD)**	0.0646	0.0660
Significant difference in sum ΔHDX	CI 99% ± 0.36 Da	

Supplementary table 4. HDX Experiment 1: tβ1AR with antagonists Carazolol and Carvedilol.

	tβ1AR apo + Cyanopindolol	*tβ1AR apo*
**HDX reaction details**	50 mM K_2HPO_4 + 50 mM KH_2PO_4 + 100 mM TCEP + 0.1% DDM	
**HDX time course (min)**	0.25, 2, 30, 120 minutes	
**Number of peptides**	205	205
**Sequence coverage**	90.06%	90.06%
**Average peptide length/redundancy**	10.67 / 6.71	10.67 / 6.71
**Replicates (technical)**	3	3
**Repeatability (average SD)**	0.0528	0.0518
Significant difference in sum ΔHDX	CI 99% ± 0.32 Da	

	**tβ1AR apo + Isoprenaline**	**tβ1AR apo**
**HDX reaction details**	50 mM K_2HPO_4 + 50 mM KH_2PO_4 + 100 mM TCEP + 0.1% DDM	
**HDX time course (min)**	0.25, 2, 30, 120 minutes	
**Number of peptides**	205	205
**Sequence coverage**	90.06%	90.06%
**Average peptide length/redundancy**	10.67 / 6.71	10.67 / 6.71
**Replicates (technical)**	3	3
**Repeatability (average SD)**	0.0542	0.0518
Significant difference in sum ΔHDX	CI 99% ± 0.31 Da	

Supplementary table 5. HDX Experiment 2: tβ1AR with antagonist Cyanopindolol and agonist Isoprenaline.

	tβ1AR apo + Norepinephrine	*tβ1AR apo*
**HDX reaction details**	50 mM K_2HPO_4 + 50 mM KH_2PO_4 + 100 mM TCEP + 0.1% DDM	
**HDX time course (min)**	0.25, 2, 30, 120 minutes	
**Number of peptides**	200	200
**Sequence coverage**	90.06%	90.06%
**Average peptide length/redundancy**	10.90 / 6.69	10.90 / 6.69
**Replicates (technical)**	3	3
**Repeatability (average SD)**	0.0678	0.0605
Significant difference in sum ΔHDX	CI 99% ± 0.37 Da	

	tβ1AR apo + Salbutamol	*tβ1AR apo*
**HDX reaction details**	50 mM K_2HPO_4 + 50 mM KH_2PO_4 + 100 mM TCEP + 0.1% DDM	
**HDX time course (min)**	0.25, 2, 30, 120 minutes	
**Number of peptides**	200	200
**Sequence coverage**	90.06%	90.06%
**Average peptide length/redundancy**	10.90 / 6.69	10.90 / 6.69

**Replicates (technical)**	3	3
**Repeatability (average SD)**	0.0658	0.0605
Significant difference in sum ΔHDX	CI 99% ± 0.36 Da	

	tβ1AR apo + Dobutamine	***tβ1AR apo***
**HDX reaction details**	50 mM K_2HPO_4 + 50 mM KH_2PO_4 + 100 mM TCEP + 0.1% DDM	
**HDX time course (min)**	0.25, 2, 30, 120 minutes	
**Number of peptides**	200	200
**Sequence coverage**	90.06%	90.06%
**Average peptide length/redundancy**	10.90 / 6.69	10.90 / 6.69
**Replicates (technical)**	3	3
**Repeatability (average SD)**	0.0600	0.0605
Significant difference in sum ΔHDX	CI 99% ± 0.35 Da	

Supplementary table 6. HDX Experiment 3: tβ1AR with agonist Norepinephrine and partial agonists Dobutamine and Salbutamol.

	tβ1AR apo + Isoprenaline	***tβ1AR apo***
**HDX reaction details**	50 mM K_2HPO_4 + 50 mM KH_2PO_4 + 100 mM TCEP + 0.1% DDM	
**HDX time course (min)**	0.25, 2 minutes	
**Number of peptides**	176	176
**Sequence coverage**	85.36%	85.36%
**Average peptide length/redundancy**	10.97 / 6.25	10.97 / 6.25
**Replicates (technical)**	3	3
**Repeatability (average SD)**	0.0444	0.0435
Significant difference in sum ΔHDX	CI 99% ± 0.30 Da	

	tβ1AR apo + Colterol HCl	***tβ1AR apo***
**HDX reaction details**	50 mM K_2HPO_4 + 50 mM KH_2PO_4 + 100 mM TCEP + 0.1% DDM	
**HDX time course (min)**	0.25, 2 minutes	
**Number of peptides**	176	176
**Sequence coverage**	85.36%	85.36%
**Average peptide length/redundancy**	10.97 / 6.25	10.97 / 6.25
**Replicates (technical)**	3	3
**Repeatability (average SD)**	0.0444	0.0435
Significant difference in sum ΔHDX	CI 99% ± 0.30 Da	

	tβ1AR apo + Orciprenaline	*tβ1AR apo*
**HDX reaction details**	50 mM K_2HPO_4 + 50 mM KH_2PO_4 + 100 mM TCEP + 0.1% DDM	
**HDX time course (min)**	0.25, 2 minutes	
**Number of peptides**	176	176
**Sequence coverage**	85.36%	85.36%
**Average peptide length/redundancy**	10.97 / 6.25	10.97 / 6.25
**Replicates (technical)**	3	3
**Repeatability (average SD)**	0.0442	0.0435
Significant difference in sum ΔHDX	CI 99% ± 0.30 Da	

Supplementary table 7. HDX Experiment 4: tβ1AR with Isoprenaline derivatives.

	tβ1ARL72A apo + Cyanopindolol	*tβ1ARL72A apo*
**HDX reaction details**	50 mM K_2HPO_4 + 50 mM KH_2PO_4 + 100 mM TCEP + 0.1% DDM	
**HDX time course (min)**	0.25, 2, 30 minutes	
**Number of peptides**	134	134
**Sequence coverage**	84.25%	84.25%
**Average peptide length/redundancy**	11.37 / 4.99	11.37 / 4.99
**Replicates (technical)**	3	3
**Repeatability (average SD)**	0.0635	0.0652
Significant difference in sum ΔHDX	CI 99% ± 0.44 Da	

	tβ1ARL72A apo + Isoprenaline	*tβ1ARL72A apo*
**HDX reaction details**	50 mM K_2HPO_4 + 50 mM KH_2PO_4 + 100 mM TCEP + 0.1% DDM	
**HDX time course (min)**	0.25, 2, 30 minutes	
**Number of peptides**	134	134
**Sequence coverage**	84.25%	84.25%
**Average peptide length/redundancy**	11.37 / 4.99	11.37 / 4.99
**Replicates (technical)**	3	3
**Repeatability (average SD)**	0.0620	0.0652
Significant difference in sum ΔHDX	CI 99% ± 0.44 Da	

Supplementary table 8. HDX Experiment 5: tβ1AR_L72A mutant with Isoprenaline and Cyanopindolol.

	tβ1AR + miniGs + Cyanopindolol	***tβ1AR apo***
**HDX reaction details**	50 mM K_2HPO_4 + 50 mM KH_2PO_4 + 100 mM TCEP + 0.1% DDM	
**HDX time course (min)**	0.03, 0.1, 0.25 minutes	
**Number of peptides**	148	148
**Sequence coverage**	87.02%	87.02%
**Average peptide length/redundancy**	10.86 / 5.10	10.86 / 5.10
**Replicates (technical)**	3	3
**Repeatability (average SD)**	0.0713	0.0599
Significant difference in sum ΔHDX	CI 99% ± 0.33 Da	

	tβ1AR + miniGs + Isoprenaline	***tβ1AR apo***
**HDX reaction details**	50 mM K_2HPO_4 + 50 mM KH_2PO_4 + 100 mM TCEP + 0.1% DDM	
**HDX time course (min)**	0.03, 0.1, 0.25 minutes	
**Number of peptides**	148	148
**Sequence coverage**	87.02%	87.02%
**Average peptide length/redundancy**	10.86 / 5.10	10.86 / 5.10
**Replicates (technical)**	3	3
**Repeatability (average SD)**	0.0598	0.0599
Significant difference in sum ΔHDX	CI 99% ± 0.34 Da	

	tβ1AR + miniGs + Dobutamine	***tβ1AR apo***
**HDX reaction details**	50 mM K_2HPO_4 + 50 mM KH_2PO_4 + 100 mM TCEP + 0.1% DDM	
**HDX time course (min)**	0.03, 0.1, 0.25 minutes	
**Number of peptides**	148	148
**Sequence coverage**	87.02%	87.02%
**Average peptide length/redundancy**	10.86 / 5.10	10.86 / 5.10
**Replicates (technical)**	3	3
**Repeatability (average SD)**	0.0595	0.0599
Significant difference in sum ΔHDX	CI 99% ± 0.36 Da	

	tβ1AR + miniGs + Cyanopindolol	**miniGs alone**
**HDX reaction details**	50 mM K_2HPO_4 + 50 mM KH_2PO_4 + 100 mM TCEP + 0.1% DDM	
**HDX time course (min)**	0.03, 0.1, 0.25 minutes	
**Number of peptides**	118	118
**Sequence coverage**	64.09%	64.09%
**Average peptide length/redundancy**	13.24 / 6.73	13.24 / 6.73
**Replicates (technical)**	3	3
**Repeatability (average SD)**	0.0815	0.0756
Significant difference in sum ΔHDX	CI 99% ± 0.58 Da	

	tβ1AR + miniGs + Isoprenaline	**miniGs alone**
**HDX reaction details**	50 mM K_2HPO_4 + 50 mM KH_2PO_4 + 100 mM TCEP + 0.1% DDM	
**HDX time course (min)**	0.03, 0.1, 0.25 minutes	
**Number of peptides**	118	118
**Sequence coverage**	64.09%	64.09%
**Average peptide length/redundancy**	13.24 / 6.73	13.24 / 6.73
**Replicates (technical)**	3	3
**Repeatability (average SD)**	0.0802	0.0756
Significant difference in sum ΔHDX	CI 99% ± 0.59 Da	

	tβ1AR + miniGs + Dobutamine	**miniGs alone**
**HDX reaction details**	50 mM K_2HPO_4 + 50 mM KH_2PO_4 + 100 mM TCEP + 0.1% DDM	
**HDX time course (min)**	0.03, 0.1, 0.25 minutes	
**Number of peptides**	118	118
**Sequence coverage**	64.09%	64.09%
**Average peptide length/redundancy**	13.24 / 6.73	13.24 / 6.73
**Replicates (technical)**	3	3
**Repeatability (average SD)**	0.0846	0.0756
Significant difference in sum ΔHDX	CI 99% ± 0.46 Da	

Supplementary table 9. HDX Experiment 6: tβ1AR in complex with miniGs and Isoprenaline, Dobutamine and Cyanopindolol.

	tβ1ARL72A + miniGs + Isoprenaline	*tβ1ARL72A alone*
**HDX reaction details**	50 mM K_2HPO_4 + 50 mM KH_2PO_4 + 100 mM TCEP + 0.1% DDM	
**HDX time course (min)**	0.03, 0.1, 0.25 minutes	
**Number of peptides**	119	119
**Sequence coverage**	84.53%	84.53%
**Average peptide length/redundancy**	11.37 / 4.42	11.37 / 4.42
**Replicates (technical)**	3	3
**Repeatability (average SD)**	0.0616	0.0563
Significant difference in sum ΔHDX	CI 99% ± 0.42 Da	

	tβ1AR + miniGs + Isoprenaline	**miniGs alone**
**HDX reaction details**	50 mM K_2HPO_4 + 50 mM KH_2PO_4 + 100 mM TCEP + 0.1% DDM	
**HDX time course (min)**	0.03, 0.1, 0.25 minutes	
**Number of peptides**	76	76
**Sequence coverage**	61.05%	61.05%
**Average peptide length/redundancy**	13.68 / 4.71	13.68 / 4.71
**Replicates (technical)**	3	3
**Repeatability (average SD)**	0.0839	0.0699
Significant difference in sum ΔHDX	CI 99% ± 0.43 Da	

Supplementary table 10. HDX Experiment 7: tβ1ARL72A mutant in complex with miniGs and Isoprenaline.

Supplementary References

1. Lau, A. M., Claesen, J., Hansen, K. & Politis, A. Deuteros 2.0: peptide-level significance testing of data from hydrogen deuterium exchange mass spectrometry. *Bioinformatics* **37**, 270–272 (2021).
2. Baker, J. G. The selectivity of beta-adrenoceptor agonists at human beta1-, beta2- and beta3-adrenoceptors. *Br. J. Pharmacol.* **160**, 1048–1061 (2010).

3. Masson, G. R. et al. Recommendations for performing, interpreting and reporting hydrogen deuterium exchange mass spectrometry (HDX-MS) experiments. *Nat. Methods* **16**, 595–602 (2019).
