# Supplementary material for: Ligand-induced conformational changes in the β1-adrenergic receptor revealed by hydrogen-deuterium exchange mass spectrometry
Source: Nat Commun. 2024 Oct 18;15:8993. doi: 10.1038/s41467-024-53161-0 (PMC11489754; doi:10.1038/s41467-024-53161-0)
Supplement: Supplementary file 3 — Description of Additional Supplementary Files [file 41467_2024_53161_MOESM3_ESM.pdf]

Description of Additional Supplementary Files

File name: Supplementary Data 1

Description: Western blot analysis of membrane preparations of CHO cells transiently transfected with either tβ1AR or L72 tβ1AR. (a) Expression was confirmed for both variants relative to transfection with empty vector, loading quantity was controlled using a BCA assay. (b) TRPC3 was also used as a loading control. (c) The crude membrane preparations (obtained following ultracentrifugation) was solubilised in both DDM and SDS, to confirm that expression yields observed are due folded protein (with only folded protein able to be solubilised by DDM.
