# Supplementary material for: Ligand-induced conformational changes in the β1-adrenergic receptor revealed by hydrogen-deuterium exchange mass spectrometry
Source: Nat Commun. 2024 Oct 18;15:8993. doi: 10.1038/s41467-024-53161-0 (PMC11489754; doi:10.1038/s41467-024-53161-0)
Supplement: Supplementary file 4 — Supplementary Data 1 [file 41467_2024_53161_MOESM4_ESM.pptx]

## Slide 1
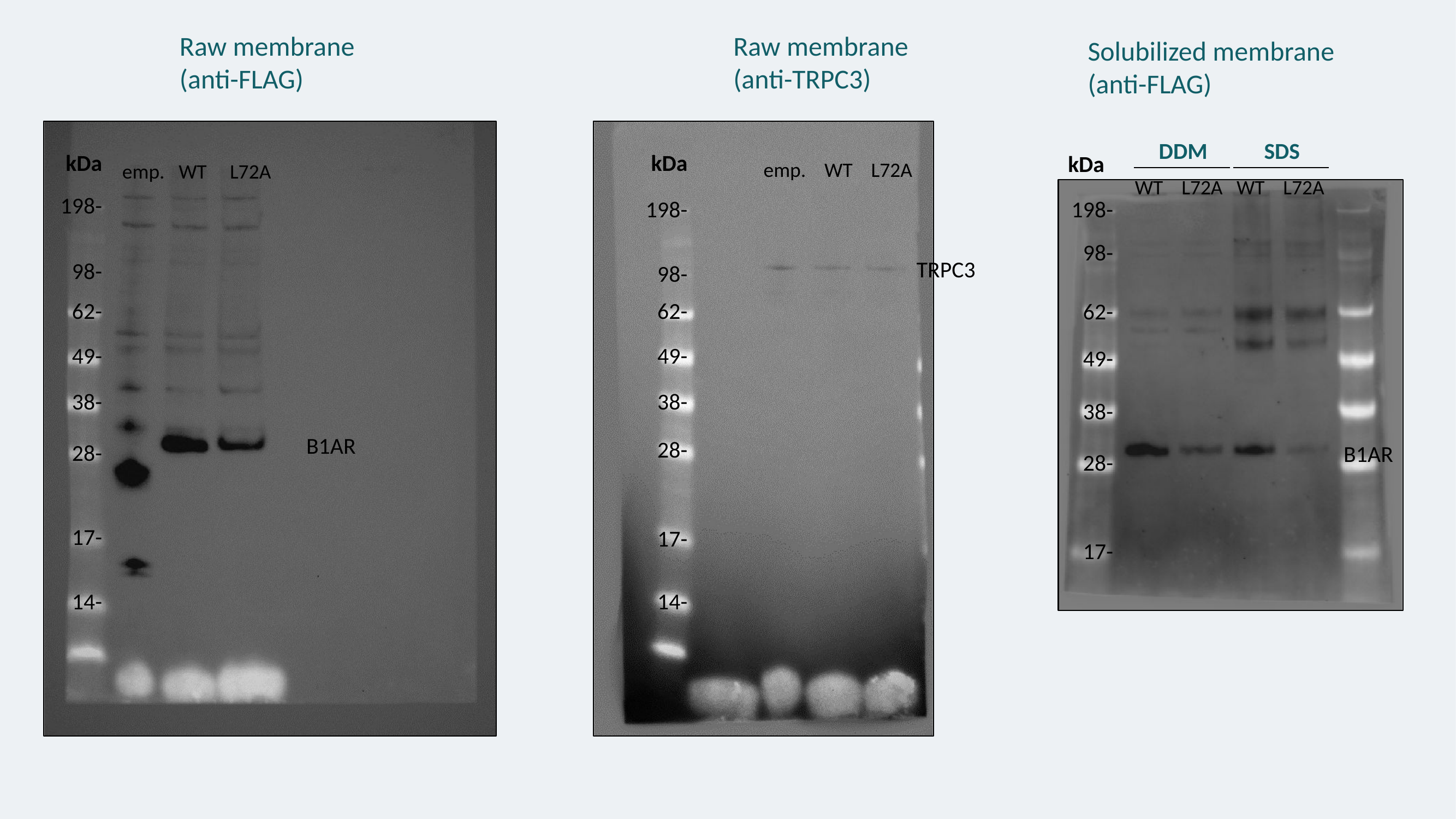

Raw membrane
(anti-FLAG)
Raw membrane
(anti-TRPC3)
Solubilized membrane
(anti-FLAG)
DDM
SDS
kDa
198-
98-
62-
49-
38-
28-
17-
14-
kDa
kDa
emp. WT L72A
emp. WT L72A
WT L72A WT L72A
198-
98-
62-
49-
38-
28-
17-
14-
198-
98-
62-
49-
38-
28-
17-
TRPC3
B1AR
B1AR
