# Supplementary material for: Ligand-induced conformational changes in the β1-adrenergic receptor revealed by hydrogen-deuterium exchange mass spectrometry
Source: Nat Commun. 2024 Oct 18;15:8993. doi: 10.1038/s41467-024-53161-0 (PMC11489754; doi:10.1038/s41467-024-53161-0)
Supplement: Supplementary file 5 — Reporting Summary [file 41467_2024_53161_MOESM5_ESM.pdf]

nature portfolio

Corresponding author(s): Argyris Politis

Last updated by author(s): 05.04.2024

Reporting Summary

Nature Portfolio wishes to improve the reproducibility of the work that we publish. This form provides structure for consistency and transparency in reporting. For further information on Nature Portfolio policies, see our Editorial Policies and the Editorial Policy Checklist.

Statistics

For all statistical analyses, confirm that the following items are present in the figure legend, table legend, main text, or Methods section.

n/a	Confirmed	
☐	☒	The exact sample size (*n*) for each experimental group/condition, given as a discrete number and unit of measurement
☐	☒	A statement on whether measurements were taken from distinct samples or whether the same sample was measured repeatedly
☐	☒	The statistical test(s) used AND whether they are one- or two-sided<br>*Only common tests should be described solely by name; describe more complex techniques in the Methods section.*
☒	☐	A description of all covariates tested
☒	☐	A description of any assumptions or corrections, such as tests of normality and adjustment for multiple comparisons
☐	☒	A full description of the statistical parameters including central tendency (e.g. means) or other basic estimates (e.g. regression coefficient) AND variation (e.g. standard deviation) or associated estimates of uncertainty (e.g. confidence intervals)
☒	☐	For null hypothesis testing, the test statistic (e.g. *F*, *t*, *r*) with confidence intervals, effect sizes, degrees of freedom and *P* value noted<br>*Give P values as exact values whenever suitable.*
☒	☐	For Bayesian analysis, information on the choice of priors and Markov chain Monte Carlo settings
☒	☐	For hierarchical and complex designs, identification of the appropriate level for tests and full reporting of outcomes
☒	☐	Estimates of effect sizes (e.g. Cohen's *d*, Pearson's *r*), indicating how they were calculated

Our web collection on statistics for biologists contains articles on many of the points above.

Software and code

Policy information about availability of computer code

Data collection	MassLynX (Waters) was used to control the Synapt G2Si mass spectrometer and to collect and save data.
Data analysis	ProteinLynx Global Server (Waters) was used to analyse the MS data of unlabelled peptide and generate peptide libraries for each target protein. DynamX (Waters) was used to analyse and quantify the deuteration for each peptide and Deuteros 2.0. was used to assess statistically significant differences in deuterium uptake for peptides in two different conditions

For manuscripts utilizing custom algorithms or software that are central to the research but not yet described in published literature, software must be made available to editors and reviewers. We strongly encourage code deposition in a community repository (e.g. GitHub). See the Nature Portfolio guidelines for submitting code & software for further information.

Data

Policy information about availability of data

All manuscripts must include a data availability statement. This statement should provide the following information, where applicable:

- Accession codes, unique identifiers, or web links for publicly available datasets
- A description of any restrictions on data availability
- For clinical datasets or third party data, please ensure that the statement adheres to our policy

The supplementary material contains the HDX summary tables for each HDX experiment conducted, as well as the deuterium uptake plots for all experiments.

Research involving human participants, their data, or biological material

Policy information about studies with human participants or human data. See also policy information about sex, gender (identity/presentation), and sexual orientation and race, ethnicity and racism.

Reporting on sex and gender	This study does not involve human research participants.
Reporting on race, ethnicity, or other socially relevant groupings	This study does not involve human research participants.
Population characteristics	This study does not involve human research participants.
Recruitment	This study does not involve human research participants.
Ethics oversight	This study does not involve human research participants.

Note that full information on the approval of the study protocol must also be provided in the manuscript.

Field-specific reporting

Please select the one below that is the best fit for your research. If you are not sure, read the appropriate sections before making your selection.

☒ Life sciences ☐ Behavioural & social sciences ☐ Ecological, evolutionary & environmental sciences

For a reference copy of the document with all sections, see nature.com/documents/nr-reporting-summary-flat.pdf

Life sciences study design

All studies must disclose on these points even when the disclosure is negative.

Sample size	Sample size calculation was deemed irrelevant for this study. Consistent with community-based recommendations (Masson et al., Nat Methods, 2019), each HDX-MS experiment was conducted with a minimum of two data points, each performed in triplicate.
Data exclusions	Peptides were omitted from analysis if their signal-to-noise ratio did not meet the criteria for precise HDX evaluation, and if the peptides lacked adequate fragmentation.
Replication	Each experiment was conducted with technical replicates, ensuring robustness. For each HDX dataset, a minimum of two data points were performed in triplicate. The summarized HDX data tables are included in the supplementary materials.
Randomization	Samples were arranged for injection based on their labeling time, with earlier time points injected before later ones. Furthermore, samples were injected in a randomized order across states for each individual data point to mitigate potential biases.
Blinding	Not relevant for this study.

Reporting for specific materials, systems and methods

We require information from authors about some types of materials, experimental systems and methods used in many studies. Here, indicate whether each material, system or method listed is relevant to your study. If you are not sure if a list item applies to your research, read the appropriate section before selecting a response.

Materials & experimental systems

n/a	Involved in the study	
☒	☐	Antibodies
☐	☒	Eukaryotic cell lines
☒	☐	Palaeontology and archaeology
☒	☐	Animals and other organisms
☒	☐	Clinical data
☒	☐	Dual use research of concern
☒	☐	Plants

Methods

n/a	Involved in the study	
☒	☐	ChIP-seq
☒	☐	Flow cytometry
☒	☐	MRI-based neuroimaging

April 2023

Eukaryotic cell lines

Policy information about cell lines and Sex and Gender in Research

Cell line source(s)	insect cells (sf9, Invitrogen, 11496015)
Authentication	cell lines were not authenticated
Mycoplasma contamination	cell lines were not tested for mycoplasma contamination
Commonly misidentified lines (See ICLAC register)	No commonly misidentified lines were used in this study

Plants

Seed stocks	not applicable for this study
Novel plant genotypes	not applicable for this study
Authentication	not applicable for this study
